# Chronic Vanadium Exposure Promotes Aggregation of Alpha‐Synuclein, Tau and Amyloid Beta in Mouse Brain

**DOI:** 10.1111/jnc.70082

**Published:** 2025-05-16

**Authors:** O. R. Folarin, F. E. Olopade, T. T. Gilbert, A. D. Ladagu, P. I. Pires dos Santos, O. A. Mustapha, L. Z. Kpasham, J. O. Olopade, T. F. Outeiro

**Affiliations:** ^1^ Department of Biomedical Laboratory Science College of Medicine, University of Ibadan Ibadan Nigeria; ^2^ Department of Anatomy College of Medicine, University of Ibadan Ibadan Nigeria; ^3^ Neuroscience Unit, Department of Veterinary Anatomy Faculty of Veterinary Medicine, University of Ibadan Ibadan Nigeria; ^4^ Department of Experimental Neurodegeneration University Medical Center Göttingen Göttingen Germany; ^5^ Department of Veterinary Anatomy College of Veterinary Medicine, Federal University of Abeokuta Abeokuta Nigeria; ^6^ Translational and Clinical Research Institute, Faculty of Medical Sciences, Newcastle University Newcastle Upon Tyne UK

**Keywords:** aging, neurodegeneration, protein aggregation, proteinopathies, vanadium

## Abstract

The interaction of toxic environmental metals and metalloids with brain proteins and polypeptides can result in pathological aggregations and formation of toxic oligomers, which are key features of many neurodegenerative diseases. Occupational and environmental exposure to vanadium is connected to a neurological syndrome that includes psychiatric symptoms, cognitive decline, and neurodegeneration. In this study, we hypothesized that prolonged vanadium exposure may be a potential risk factor for Alzheimer's and Parkinson's diseases. A total of 72 male BALB/c mice, each 4 weeks' old, were used. Vanadium‐treated groups received intraperitoneal injections of 3 mg/kg body weight of vanadium three times a week for 6, 12, or 18 months. The control group received sterile water while the withdrawal group were given vanadium injection for 3 months, followed by withdrawal of the metal, but treatment with sterile water only, and were sacrificed at 3‐, 9‐, or 15‐months post vanadium exposure. Sagittal sections of brain paraffin‐embedded tissue were prepared and analyzed using immunofluorescence to study the immunoreactivity and cellular localization of α‐synuclein (α‐syn), amyloid‐β (Aβ), and tau proteins. Our findings revealed pathological aggregation of these proteins in the frontoparietal cortices and the dorsal CA1 and CA3 regions. Double immunolabeling with glial cells and neurons showed neuronal degeneration, functional gliosis, and activation of astrocytes and microglia at sites of α‐synuclein immunoreactivity. We observed increased immunoreactivity of phosphorylated nuclei tau both in the parietal cortices and corpus callosum white matter while we observed intraneuronal accumulation of Aβ deposits in the cortex and dorsal hippocampal regions in vanadium treated brains. These cellular changes and proteinopathies, although persisting, were significantly attenuated after vanadium withdrawal. Our findings show that prolonged vanadium exposure promotes abnormal accumulation of neurodegeneration‐associated proteins (α‐syn, Tau, and Aβ) in the brain, which is further exacerbated by aging in the context of extended exposure to the metal.

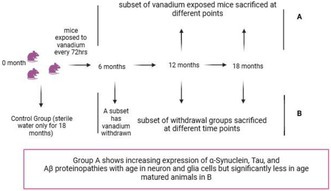

## Graphical abstract

Mice were exposed to the environmental neurotoxicant‐Vanadium periodically till old age (18 months). The animals were sacrificed at different time points to assess if this prolonged exposure is a risk for developing Alzheimer's and Parkinson's disease lesions. Our findings show that vanadium exposure promotes the accumulation of neurodegeneration‐associated proteins (α‐synuclein (α‐syn), amyloid‐β (Aβ) and tau proteins) from middle age, which become further exacerbated with aging as the exposure continues. These pathological protein accumulations were significantly attenuated after vanadium withdrawal at 3 months. Vanadium exposure is a risk factor for neurodegenerative diseases and old age dementia.

**Abbreviations**
α‐syn: Alpha‐synuclein
Aβ: amyloid beta
AD: Alzheimer's disease
ANOVA: analysis of variance
b.w.: body weight
BSA: bovine serum albumin
CA1: cornu ammonis area 1
Ca^2+^
: calcium ion
CA3: cornu ammonis area 3
CNS: central nervous system
CSF: cerebrospinal fluid
DAPI: 4′,6‐diamidino‐2‐phenylindole
DRP1: dynamin‐related protein 1
GFAP: glial fibrillary acidic protein
i.p.: intraperitoneal
Iba1: ionized calcium‐binding adaptor molecule 1
ISF: interstitial fluid
MARK: mitogen‐activated protein kinase
mg/kg: milligram per kilogram
*n*
: number of animals per group
NeuN: neuronal nuclei
NFTs: neurofibrillary tangles
PBS: phosphate buffer saline
PD: Parkinson's disease
PINK‐1: PTEN‐induced kinase 1
PKC δ: protein kinase C delta
PTEN: phosphatase and TENsin
ROS: reactive oxygen species
UI‐ACUREC: University of Ibadan Animal Care and Use Research Ethics Committee

## 1 Introduction

Vanadium is a metalloid ubiquitously found in nature (Olaolorun et al. 2021; Gilbert et al. 2023); its release into the environment occurs mainly through human activities such as heavy metal mining, crude oil combustion, and industrial processes. This presents a threat to the general populace, especially to people residing in oil‐producing regions like the Nigerian Niger Delta (Olopade and Connor 2011; Usende et al. 2022), in Jeddah, Saudi Arabia (Khodeir et al. 2012), and in industrial settings (Trovo et al. 2019), where individuals are occupationally and environmentally exposed over time. Vanadium produces oxidative damage in biological systems through the generation of free radicals and oxidative stress via Fenton‐type reactions (Fatola et al. 2019). Some of the pathological effects of vanadium in the brain include myelin damage, neuroinflammation, and neuro‐behavioral and neurochemical alterations (Zwolak et al. 2019; Folarin et al. 2016; Folarin, Snyder, et al. 2017). Other vanadium‐induced neuropathologies known are disruption of the blood‐brain barrier, dendritic spine loss, cognitive impairments, and motor impairments (Olopade and Connor 2011). In recent times, chronic metal toxicity has been strongly linked with an increased risk for the onset and progression of many neurodegenerative diseases (Folarin, Snyder, et al. 2017; Engwa et al. 2019; Shvachiy et al. 2023). Oxidative stress, neuronal loss, synaptic alterations, neuroinflammation, cognitive deficits, and dyshomeostasis of bio‐metals leading to protein misfolding and pathologic aggregation have been reported as the major consequences (Farina et al. 2013; Sweeney et al. 2017; Zwolak et al. 2019; Zwolak 2020; Folarin et al. 2021; Lamptey et al. 2022; Alamri et al. 2023; Ladagu et al. 2023). Pathologic protein aggregation and deposition are common features of all age‐related neurodegenerative diseases and, therefore, constitute a pathological hallmark of the disorders (Chiti and Dobson 2006; Leal et al. 2012). Although mutations favor protein aggregation, labile metal ions within the cellular environment also play an essential role (Leal et al. 2012). There is accumulating evidence that metals might enhance the aggregation propensity of disease‐associated proteins through largely unknown mechanisms (Ibstedt et al. 2014; Tamás et al. 2014). In the pathophysiological mechanisms of many neurodegenerative diseases, altered metal homeostasis and metal–polypeptide interactions (metal binding/release) have been implicated as two key mediators of protein oligomerization and aggregation into toxic forms (Leal et al. 2012).

Several studies investigated the interaction of metals with the aggregation of proteins and polypeptides (alpha‐synuclein (α‐syn), tau, or amyloid‐β (Aβ) peptide) into toxic metal‐oxidized protein assemblies (Miller et al. 2012); for example, binding of copper and iron to α‐syn leads to the formation of molten globule type conformers, which results in an increased aggregation rate (Santner and Uversky 2010). Environmental exposure to lead, mercury, and aluminum is linked with metal ions dyshomeostasis and toxic aggregation of α‐syn in Parkinson's disease (PD) pathogenesis (Raj et al. 2021; Ullah et al. 2021). Also, exposure to lead, manganese, aluminum, cadmium and arsenic increased the accumulation of Alzheimer's Disease (AD)‐like pathologies such as senile/amyloid plaques and neurofibrillary tangles in the brain (Myhre et al. 2013; Chin‐Chan et al. 2015). Binding of aluminum, iron, copper, zinc, cadmium, and mercury to tau results in pathologic aggregation in the brain (Ma et al. 2013; Soragni et al. 2008; Bader et al. 2011; Yang et al. 2012). In addition, epigenetic effects due to chronic metals exposure may generate genetic aberration leading to phenotypic diversity, which may induce mutagenic toxic aggregation of proteins and enhance susceptibility to neurodegeneration (Chin‐Chan et al. 2015; Khalid and Abdollahi 2019).

However, there is a dearth of information in the literature that links long‐term vanadium exposure as a risk factor for AD and PD. Therefore, in this present study, we aimed at determining whether chronic exposure to vanadium from early life would enhance the expression of toxic misfolded protein aggregates, including hyperphosphorylated tau, amyloid plaques, and α‐syn in different brain regions later in adult life.

## 2 Materials and Methods

### 2.1 Ethics Statement

All experimental procedures involving animals were approved and carried out in accordance with the guidelines by the Institutional Animal Care and Use Committee of the University of Ibadan, ethical code number UI‐ACUREC/App/2016/011.

### 2.2 Animal Treatment

A total of 72 male BALB/c mice (4 weeks old, mean weight 16.57 ± 0.37 g) were obtained from the Institute for Advanced Medical Research and Training (IAMRAT), University College Hospital (UCH), Ibadan. The experiment lasted for 18 months. No formal randomization method or sample size calculation was applied for group allocation. Female mice were excluded from the study based on exclusion criteria. The animals were arbitrarily assigned to one of the following treatment groups: Vanadium (sodium metavanadate)‐treated (V), Control (C), and Withdrawal (W) groups following the method described by Folarin, Snyder, et al. (2017). Each group consisted of eight male mice sourced from two to three different dams. In total, the study comprised nine distinct animal groups, each containing eight companions (*n* = 8). The mice were bred and housed in plastic cages (dimensions: length = 43 cm, width = 30 cm, height = 26 cm) within the experimental animal facility of the Neuroscience Unit, Department of Veterinary Anatomy, University of Ibadan. They had ad libitum access to food and water and were maintained under a 12‐h light/dark cycle.

### 2.3 Experimental Design

Vanadium (V)‐treated group composed of 3 subgroups of animals based on months of treatment. The subgroups are represented by V6, V12, and V18, *n* = 8. The mice (from 4 weeks of age) were intraperitoneally (i.p.) administered 3 mg/kg b.w./day of vanadium (sodium metavanadate, Sigma‐Aldrich, St. Louis, MO, USA), thrice a week for 6, 12, and 18 months. The dose and route of administration used for the experiment were described by García et al. (2004). Age matched control group received volume matched with sterile water using the same route and designated as C6, C12, and C18. Withdrawal group consisted of 3 subgroups of 8 animals (W3, W9 and W15); here the mice (from 4 weeks old) were i.p. administered 3 mg/kg b.w./day of vanadium (sodium metavanadate Sigma‐Aldrich, St. Louis, MO, USA), i.p. thrice a week only for 3 months and then vanadium administration stopped. Subsequently, the animals were treated with sterile water as controls and sacrificed after withdrawal from treatment at 3 months, 9 months, and 15 months post‐exposure.

### 2.4 Sample Collection

Eight (8) animals from each group were sacrificed (based on Folarin, Snyder, et al. 2017), 24 h post‐exposure. At the time of sacrifice, the mice were deeply anesthetized (ketamine 100 mg/kg and xylazine 10 mg/kg, i.p) and then transcardially perfused with phosphate buffered saline (PBS) followed by 4% paraformaldehyde in PBS (0.1 mol/L PBS, pH 7.4) with the aid of a perfusion pressure pump. Subsequently, brains were removed, post‐fixed for 24 h in 4% paraformaldehyde, and embedded in paraffin wax as described by Folarin, Snyder, et al. (2017). Mid‐sagittal sections were cut from paraffin‐embedded tissue on a standard microtome at 5 mm thickness. Sections were collected on Super FrostPlus Slides (J1800AMNZ, Thermo Fisher Scientific Inc., Germany).

### 2.5 Immunostaining and Immunofluorescence

Paraffin sections were dewaxed in xylene (Cat# 108298, Sigma‐Aldrich, Germany), rehydrated in graded ethanol (Cat# 108543, Sigma‐Aldrich, Germany) solutions, and immersed in distilled water. Antigen retrieval was performed by heating for 25mins in 10 mM citrate buffer (pH 6.0; Cat# H‐3300, Vector Laboratories, United State), after washing in distilled water, sections were permeabilized for 10 min with 0.3% Triton‐X (Cat# 9002931; Sigma‐Aldrich, Germany) in PBS 1X (mixing 100 mL of PBS 10X (14190‐250, Thermo Fisher Scientific inc) with 900 mL d‐H_2_O), blocked for 1 h with 5% BSA (Cat# A 9647, Sigma‐Aldrich, Germany) in 0.3% PBS Triton‐X, and incubated overnight (an added 24 h for Iba‐1 antibody) at 4°C with the following antibodies (Table 1): anti‐GFAP Chicken polyclonal antibody for astrocytic morphology (Abcam Cat# ab4674, RRID: AB_304558), (Abcam Cat# ab104225, RRID: AB_10711153), and (Abcam Cat# ab104224, RRID: AB_10711040) for neuronal morphology; anti‐Iba‐1 Goat polyclonal antibody for microglia morphology (Abcam Cat# ab5076, RRID: AB_2224402); primary antibodies specific for alpha synuclein (BD Biosciences Cat# 610787, RRID: AB_398108), Amyloid beta 1‐42 fibrils (Abcam Cat# ab201062, RRID: AB_3674664) and Tau (Abcam Cat# ab32057, RRID: AB_778254) were also diluted in 1% BSA in 0.3% PBS Triton‐X for 16 h at 4°C and subsequently washed in PBS 1X. For double immuno‐labeling, sections were incubated at room temperature for 1 h with a mixture of fluorescent‐conjugated secondary antibodies donkey anti‐mouse Alexa Fluor 488 (Invitrogen Cat# A21202), donkey anti‐rabbit Alexa Fluor 488 (Invitrogen Cat# A21206), goat anti‐rabbit Alexa Fluor 568 (Invitrogen Cat# A11011); goat anti‐chicken Alexa Fluor 633 (Invitrogen, Cat# A21103), donkey anti‐goat Alexa Fluor 633 (Invitrogen Cat# A21082) antibodies diluted at 1:400 in 1% BSA in 0.03% PBS Triton‐X. To identify nuclear DNA in the cell types, sections were incubated for 5 min with 40,6‐diamidine‐20‐pheynylindole dihydrochloride, 1:1000 (DAPI; Invitrogen; Cat# EN62248). After a final wash, slides were mounted in Fluoromount Aqueous Mounting Medium (Cat# F4680, Sigma‐Aldrich, Germany), allowed to dry for 24 h at 23°C–26°C, protected from light exposure, and subjected to fluorescence microscopy. Z‐stack images at 20× magnification were acquired with a Zeiss Axio Observer Widefield Fluorescence Microscope (Cat No: FFSC 4226) connected to a digital camera Hamamatsu orca‐R2 (C10600‐10B) (1344 × 1024).

**TABLE 1:** List of the primary and secondary antibodies used for immunofluorescence staining.

Serial no	Names	Source	Catalog No.	RRID no	Dilution
1	Chicken anti‐GFAP	Abcam, Cambridge, United Kingdom	ab4674	AB_304558	1:1000
2	Rabbit anti‐NeuN	Abcam, Cambridge, United Kingdom	ab104225	AB_10711153	1:500
3	Mouse anti‐NeuN	Abcam, Cambridge, United Kingdom	ab104224	AB_10711040	1:500
4	Mouse anti‐ α‐syn	BD Biosciences, San Diego, USA	610 787	AB_398108	1:200
5	Rabbit anti‐Amyloid Fibril antibody [mOC87]	Abcam, Cambridge, United Kingdom	ab201062	AB_3674664	1:1000
6	Rabbit Anti‐tau	Abcam, Cambridge, United Kingdom	ab32057	AB_778254	1:1000
7	Goat anti‐Iba 1	Abcam, Cambridge, United Kingdom	ab5076		1:200
8	Goat Alexa Fluor 633‐conjugated anti‐chicken (IgG)	Invitrogen, Darmstadt, Germany	A21103		1:400
9	Goat Alexa Fluor 568‐conjugated anti‐rabbit (IgG)	Invitrogen, Darmstadt, Germany	A11011		1:400
10	Donkey Alexa Fluor 488‐conjugated anti‐mouse (IgG)	Invitrogen, Darmstadt, Germany	A21202		1:400
11	Donkey Alexa Fluor 633‐conjugated anti‐goat (IgG)	Invitrogen, Darmstadt, Germany	A21447		1:400
12	Donkey Alexa Fluor 488‐conjugated anti‐rabbit (IgG)	Invitrogen, Darmstadt, Germany	A21206		1:400

### 2.6 Microscopy and Image Analysis

For the qualitative and quantitative evaluation, all image analyses were performed by one blinded observer to avoid experimental bias. Z‐stack images at 20× magnification were captured from the frontoparietal cortex, dorsal CA1 and CA3 regions, and the genu of the corpus callosum with a Zeiss Axio Observer Widefield Fluorescence Microscope equipped with 5×, 10×, 20× dry, and 63×/100× oil objectives connected to a digital camera Hamamatsu orca‐R2 (C10600‐10B) (1344 × 1024). Before image collection, image and laser settings that could affect image intensity and brightness were standardized to a control. Identical light intensity and exposure settings were applied to all images taken for each experimental set. Quantification was carried out on 6 brain samples using Image‐J plug‐in software (NIH). Data were further analyzed statistically using GraphPad Prism 7.

### 2.7 Stereological Analysis

#### 2.7.1 Quantification of Neurons, Astrocytes, Microglia and Tau Immunostaining

For the evaluation of neurons (prefrontal and hippocampal region of CA1 and CA3), astrocytes, and microglia (CA1 and CA3 and frontoparietal cortex) immunostaining was carried out, and cells within the sampled region were counted and evaluated. Tau immunoreactive cells within the frontoparietal cortices and genu of the corpus callosum were also counted and analyzed.

Three arbitrary images from each section (sections per mouse; *n* = 6) of these immunoreactive cells (neurons, astrocytes, microglia) in the frontoparietal cortices, dorsal hippocampal CA1, and hippocampal CA3 regions respectively were acquired at 20× magnification. In each section, a region was defined within one 20× field that corresponded to ~460 μm. Positively stained cells were counted in three comparable, randomly selected 20× microscopic fields. The numbers of immunoreactive cells from three locations per mouse (3 fields/section × 2 sections/mouse) were averaged and expressed as positive cells per field (20×).

#### 2.7.2 Evaluation of Alpha‐Synuclein and Amyloids Staining Intensity

For the quantification of the alpha‐synuclein and amyloids staining intensity, using the afore‐described sampling method, six photographs each were taken from the frontoparietal cortex, dorsal CA1, and CA3. Densitometric quantification of alpha‐synuclein and amyloid immunoreactivity was determined and normalized to the corresponding control using Image‐J plug‐in fiji software.

### 2.8 Statistical Analysis

All data were presented as mean ± standard deviation. Normality was assessed prior to analysis to determine suitability for parametric testing using the Shapiro–Wilk test. Normally distributed data were analyzed using one‐way ANOVA, followed by Tukey's post hoc test for group comparisons. Statistical significance was set at *p* < 0.05. No outlier tests were conducted. All statistical analyses were performed using GraphPad Prism Version 7 (GraphPad Software, San Diego, CA, USA). Statistical reports of data set are provided in Table 2.

**TABLE 2:** Statistical reports of data set showing degrees of freedom, *F* value, actual *p* value.

Parameters	Fronto‐parietal cortex (FPC)			CA1 hippocampus			CA3 hippocampus		
	6 m	12 m	18 m	6 m	12 m	18 m	6 m	12 m	18 m
Astrocytic count									
Degrees of freedom (DF)	17	17	17	17	17	17	17	17	17
*F* value (*F*)	1	3	4	4	6	2	3	1	1
Actual *p* value	*p* < 0.0001	*p* < 0.0001	*p* < 0.0001	*p* < 0.0001	*p* < 0.0001	*p* < 0.0001	*p* < 0.0001	*p* < 0.0001	*p* < 0.0001
Microglial count									
Degrees of freedom (DF)	17	17	17	17	17	17	17	17	17
*F* value (*F*)	5	4	5	6	1	1	5	6	6
Actual *p* value	*p* < 0.0001	*p* < 0.0001	*p* < 0.0001	*p* < 0.0001	*p* < 0.0001	*p* < 0.0001	*p* < 0.0001	*p* < 0.0001	*p* < 0.0001
Neuronal count									
Degrees of freedom (DF)	17	17	17	17	17	17	17	17	17
*F* value (*F*)	109.5	229.7	90.88	58.78	42.77	23.83	27.39	76.04	73.50
Actual *p* value	*p* < 0.0001	*p* < 0.0001	*p* < 0.0001	*p* < 0.0001	*p* < 0.0001	*p* < 0.0001	*p* < 0.0001	*p* < 0.0001	*p* < 0.0001
Alpha synuclein intensity									
Degrees of freedom (DF)	17	17	17	17	17	17	17	17	17
*F* value (*F*)		51.11	1	127.6	81.91	154.6	1	7	3
Actual *p* value	*p* < 0.0001	*p* < 0.0001	*p* < 0.0001	*p* < 0.0001	*p* < 0.0001	*p* < 0.0001	*p* < 0.0001	*p* < 0.0001	*p* < 0.0001
Amyloid beta intensity									
Degrees of freedom (DF)	17	17	17	17	17	17	17	17	17
*F* value (*F*)	191.5	229.8	104.6	136.8	1	3	7	175.4	379.2
Actual *p* value	*p* < 0.0001	*p* < 0.0001	*p* < 0.0001	*p* < 0.0001	*p* < 0.0001	*p* < 0.0001	*p* < 0.0001	*p* < 0.0001	*p* < 0.0001
Tau intensity									
FPC				Corpus callosum					
Degrees of freedom (DF)	17	17	17	17	17	17			
*F* value (*F*)	115.3	186.6	330.5	40.38	93.74	95.74			
Actual *p* value	*p* < 0.0001	*p* < 0.0001	*p* < 0.0001	*p* < 0.0001	*p* < 0.0001	*p* < 0.0001			

## 3 Results

### 3.1 Astrocytic Activation After Chronic Vanadium Exposure and Withdrawal

First, we assessed the effect of vanadium exposure on astrocytes. Immunohistochemical identification and characterization of astrocytes was carried out using GFAP and revealed notable upregulation of GFAP in astrocytic cells along with hypertrophied somata and well‐ramified cytoplasmic processes in the vanadium‐treated group. Conversely, control tissue displayed astrocytes in a quiescent state, characterized by their resting morphology. Tissue from mice that underwent withdrawal from vanadium exhibited a notable reduction in astrocytic activation compared to that from animals chronically exposed to vanadium. Moreover, these observations were further corroborated with quantitative analysis of astrocytes across groups, with vanadium‐dosed groups consistently having higher astrocytic counts compared to control and withdrawal groups of the same time points. Intriguingly, we observed a progressive increase in astrogliosis in vanadium‐exposed animals during the initial exposure period of 6–12 months, followed by a gradual attenuation of astrogliosis from 12 to 18 months (Figures S1B, S2B and S3B).

### 3.2 Microglia Activation After Chronic Vanadium Exposure and Withdrawal

Using Iba1, we demonstrated and characterized the morphological phenotypes, density, and activation status of microglia across groups. In the vanadium‐treated groups, microglial activation characterized by a progressive increase in the number of Iba1‐positive cells with morphologies leaning more towards phagocytic phenotypes was observed in the hippocampal CA1 and CA3 regions, as well as in the frontoparietal cortices compared to their matched control group. Typically, in these brain regions, a progressive reduction/shrinkage of microglial processes highlighted by their short, stout processes was observed in mice exposed to vanadium with chronicity. The brains of mice in the withdrawal group showed reduced Iba1 immunoreactivity compared to those continuously exposed to vanadium. Furthermore, quantitative analysis indicated a progressive increase in microgliosis in brains exposed to vanadium over the duration of the exposure period, spanning from 6 to 18 months (Figures S1C, S2C and S3C).

### 3.3 Neuronal Loss After Progressive Vanadium Exposure and Withdrawal

Next, immunohistochemical evaluation using NeuN, a neuron‐specific marker, was carried out across the groups. In the vanadium‐exposed group, a decrease in immunostaining of NeuN‐positive cells was noted both in the frontoparietal cortex and hippocampus, whereas in the matched controls, an increase in immunoreactive‐NeuN cells was observed. Mice withdrawn from vanadium treatment showed a potential recovery and increased immunoreactivity to NeuN compared to mice that were chronically exposed to vanadium. Correspondingly, quantitative analysis showed similar observations with a progressive decline in neuronal cell counts in brain regions of mice dosed with vanadium during the initial exposure period from 6 to 18 months compared to matched controls and withdrawal groups (Figures S1A, S2A and S3A).

### 3.4 α‐Syn Immunolabeling and Cellular Localization

**FIGURE 1:**
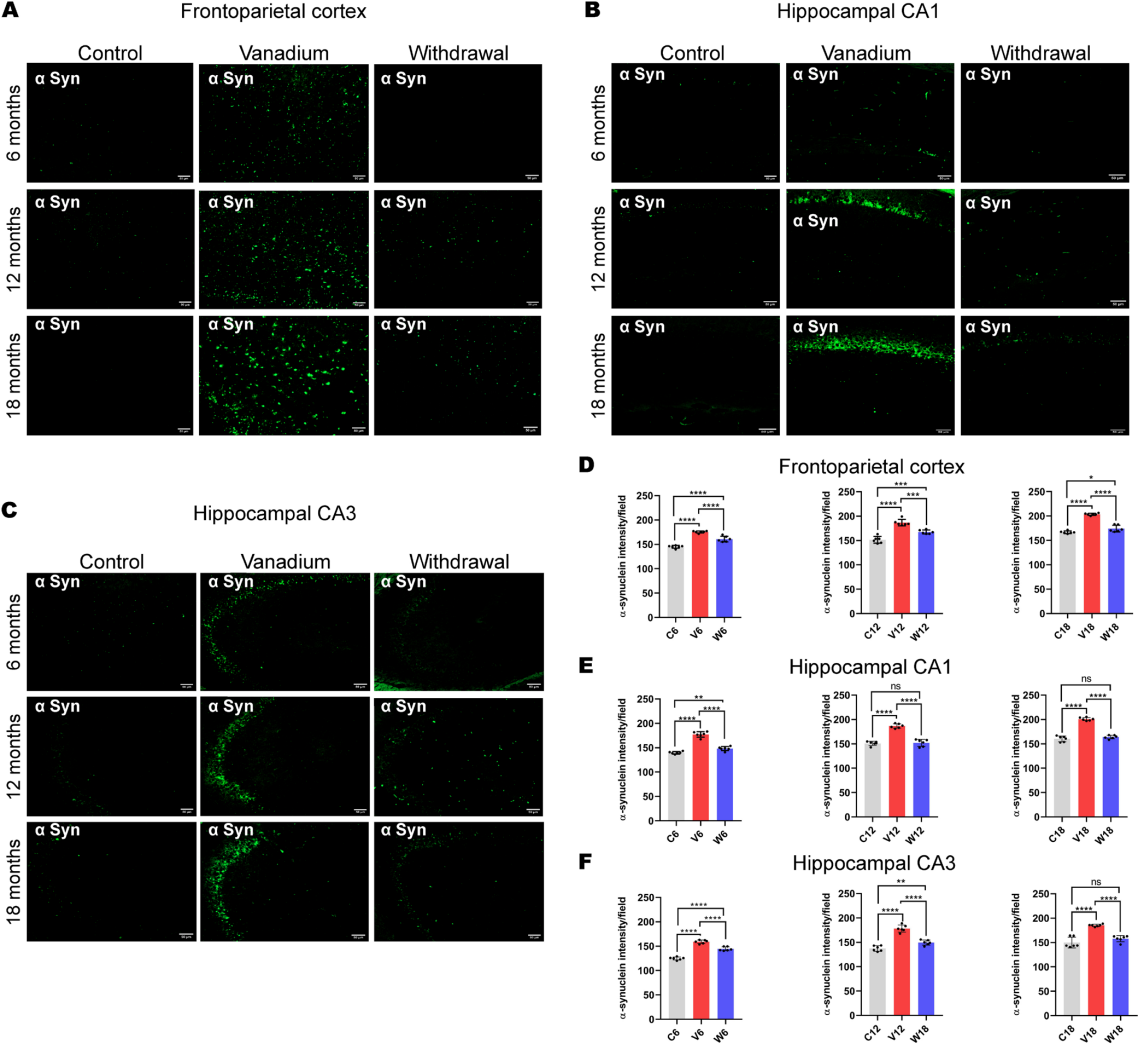
Immunofluorescent staining showing the cellular localization of alpha‐synuclein (α‐syn) in the mice brains following chronic vanadium exposure and subsequent withdrawal. α‐Syn aggregation was detected in the frontoparietal cortices, FPC (A), hippocampal CA1 (B), and hippocampal CA3 (C) after chronic vanadium treatments. At each exposure period (6, 12, and 18 months), there was a significant increase in α‐syn aggregation in vanadium exposed compared to controls. However, a reduction was observed in the withdrawal brains compared to vanadium exposed brains. Densitometric quantification confirmed a trend of increasing α‐syn aggregation with advancing age and the duration of vanadium exposure. (D): α‐syn expressions in the cortex at 6, 12, and 18 months respectively; (E, F): Mean α‐syn expressions in the hippocampus (CA1 and CA3) at 6, 12, and 18 months respectively. [*n* = 8 mice/group; intensity analysis = 6 mice; Scale bar: 50 μm; *p* > 0.05; Normality test (Shapiro–Wilk) = FPC (6 months: *p* value 0.8498; 12 months: *p* value 0.0758; 18 months: *p* value 0.7509). CA1 (6 months: *p* value 0.1519; 12 months: *p* value 0.2002; 18 months: *p* value 0.48811). CA3 (6 months: *p* value 0.0926; 12 months: *p* value 0.6613; 18 months: *p* value 0.1903) (*p < 0.05; **p < 0.01; ***p < 0.001; ****p < 0.0001)].

**FIGURE 2:**
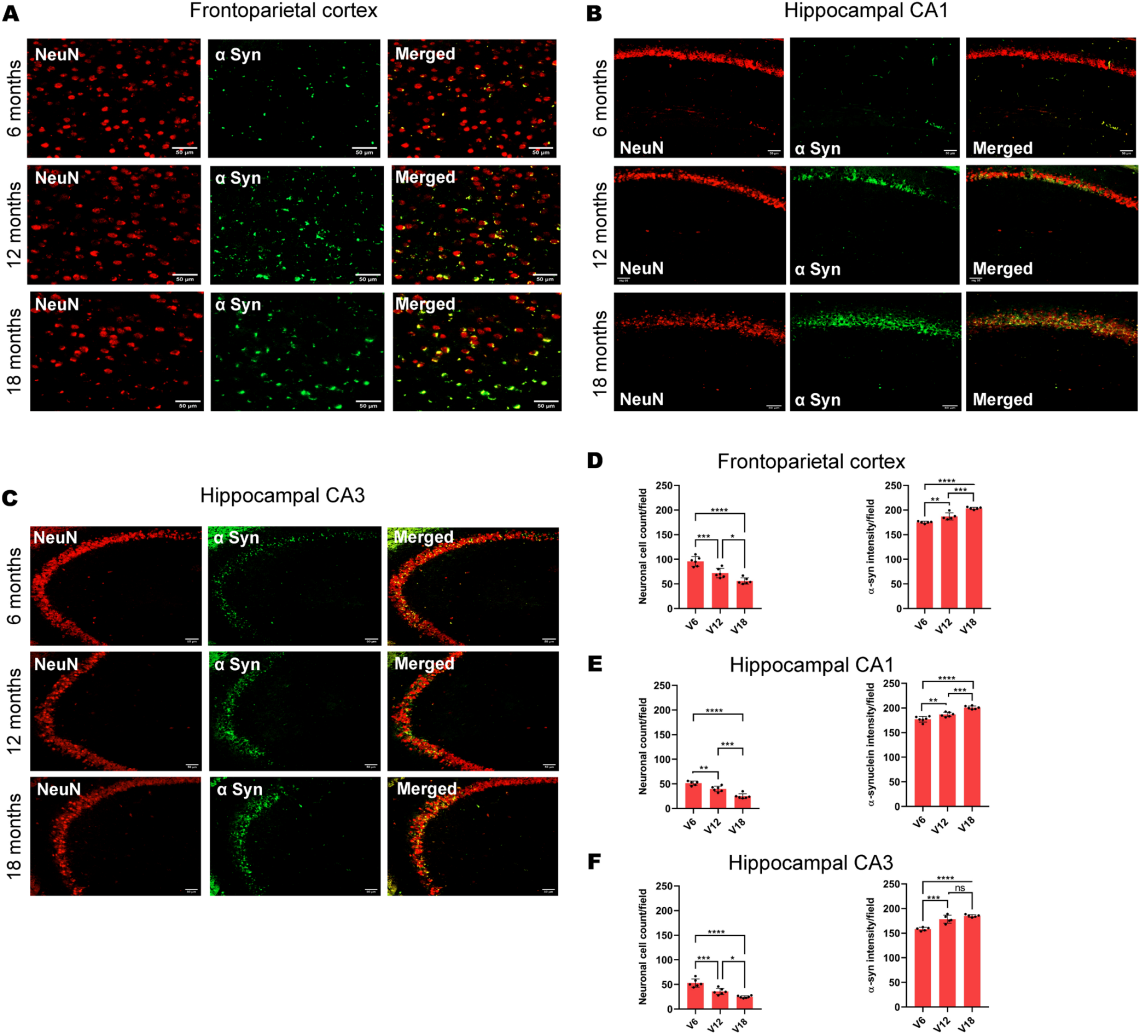
NeuN/α‐syn double immunolabeling of the mice brains following chronic vanadium exposure revealed alpha‐synuclein (α‐syn) positive aggregates in the frontoparietal cortical (FPC) pyramidal cells (A), hippocampal CA1 region (B), and hippocampal CA3 region (C). The level of α‐syn positive protein aggregates increased progressively from 6 to 18 months of vanadium exposure (A–C), which strongly correlated with the significant neuropathology observed in these regions. Colocalization of α‐syn immunolabeling with NeuN indicated neuronal pathology characterized by the accumulation of α‐syn positive aggregates and cytoplasmic toxicity in cortical pyramidal cells. Quantification of neuronal count indicated marked cell loss that increased with the chronicity of vanadium exposure in the cortex (D) and hippocampus (E, F). [*n* = 8 mice/group; intensity analysis = 6 mice; Scale bar: 50 μm; *p* > 0.05; Normality test (Shapiro–Wilk) = Neuronal loss (FPC: *p* value 0.3565, CA1: *p* value 0.1365, CA3: *p* value 0.2838); α‐syn aggregation (FPC: *p* value 0.0185, CA1: *p* value 0.5664, CA3: *p* value 0.9021) (*p < 0.05; **p < 0.01; ***p < 0.001; ****p < 0.0001)].

**FIGURE 3:**
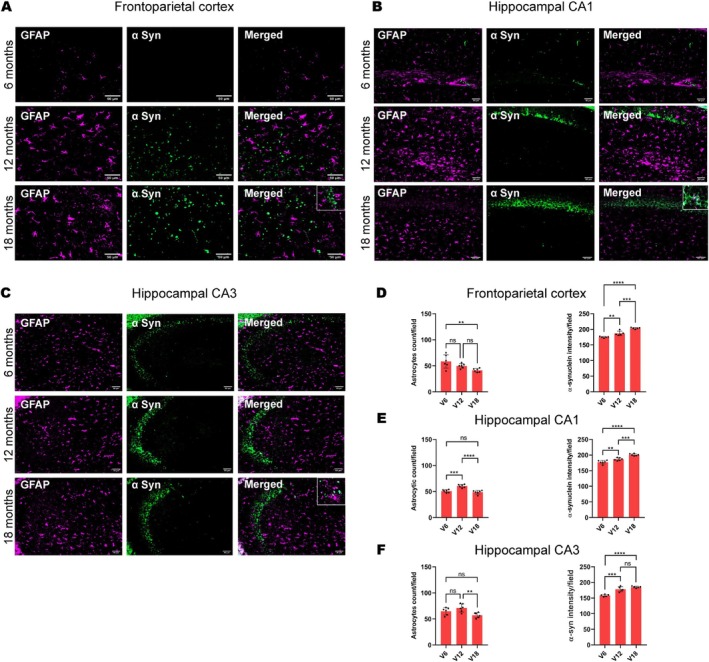
GFAP/α‐syn double immunolabeling of the mice brains after chronic vanadium exposure revealed astrocytic activation in the frontoparietal cortical (FPC) pyramidal cells (A), hippocampal CA1 region (B), and hippocampal CA3 region (C). Astrogliosis increased progressively from 6 to 18 months of vanadium exposure (A–C), with corresponding increase in α‐syn immunoreactivity in these regions. Colocalization of α‐syn immunolabeling with the astrocytic marker glial fibrillary acidic protein (GFAP) revealed the presence of α‐syn positive aggregates in activated astrocytes (see insets). Quantitative analysis of astrocyte counts and α‐syn protein aggregation showed significant activation, correlating α‐syn protein aggregation with the chronicity of vanadium exposure in the cortex (D) and hippocampus (E, F). [*n* = 8 mice/group; intensity analysis = 6 mice; Scale bar: 50 μm, *p* > 0.05; Normality test (Shapiro–Wilk) = Astrocytic count (FPC: *p* value 0.4718, CA1: *p* value 0.1239, CA3: *p* value 0.3329), α‐syn (FPC: *p* value 0.0185, CA1: *p* value 0.5664, CA3: *p* value 0.9021) (**p < 0.01; ***p < 0.001; ****p < 0.0001)].

**FIGURE 4:**
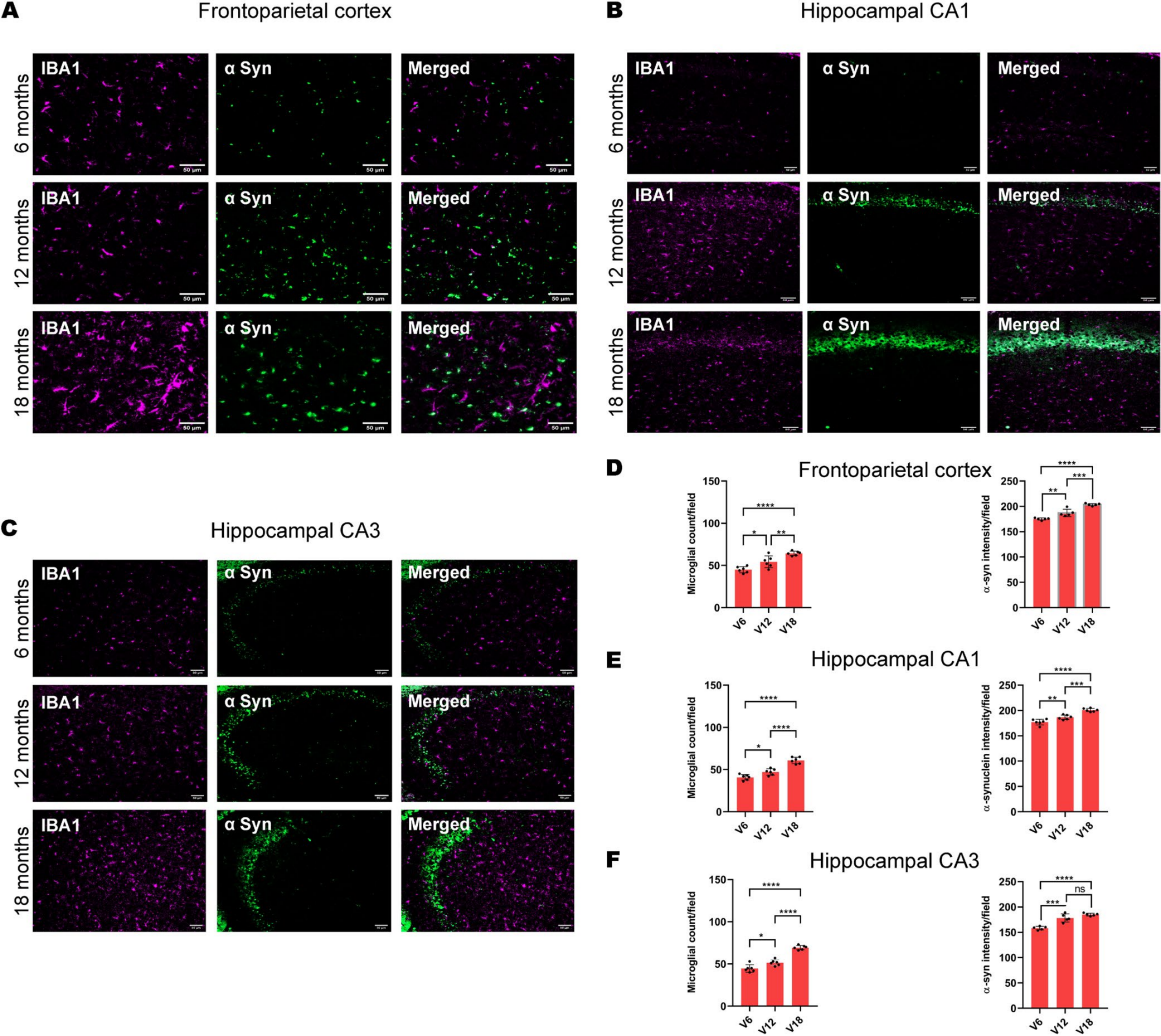
IBA1/α‐syn double immunolabeling of the mouse brain after chronic vanadium exposure revealed microglial activation in the frontoparietal cortical (FPC) pyramidal cells (A), hippocampal CA1 region (B), and hippocampal CA3 region (C). Microglial activation increased progressively from 6 to 18 months of vanadium exposure (A–C), with a corresponding increase in α‐syn immunoreactivity observed in these regions. Colocalization of α‐syn immunolabeling with the microglial marker ionized calcium‐binding adapter molecule 1 (IBA1) revealed α‐syn positive microglia and phagocytosis (see insets). Quantitative analysis indicated significant microglial activation and α‐syn protein aggregation with chronicity of vanadium exposure in the cortex (D) and hippocampus (E, F). [*n* = 8 mice/group; intensity analysis = 6 mice; Scale bar: 50 μm; *p* > 0.05; Normality test (Shapiro–Wilk) = Microglial count (FPC: *p* value 0.9358, CA1: *p* value 0.1385, CA3: *p* value 0.2707), α‐syn (FPC: *p* value 0.0185, CA1: *p* value 0.5664, CA3: *p* value 0.9021) (*p < 0.05; **p < 0.01; ***p < 0.001; ****p < 0.0001)].

Animals treated with vanadium for 6–18 months had increased expressions of intra‐cellular α‐syn aggregation which progressed with chronicity of treatment (Figure 1). Control mice brains had little or no α‐syn aggregation in their frontoparietal cortices (Figure 1A) and dorsal hippocampal CA1 (Figure 1B) and CA3 regions (Figure 1C). There was a significant difference in the percentage of cells displaying α‐syn inclusions between exposed and control animals (Table 2). However, withdrawal of vanadium resulted in some degree of attenuation in α‐syn immunoreactivity relative to the exposed animals (Figure 4A–C, Panels 3). This result also showed that α‐syn aggregation in the vanadium‐exposed brains increases with increasing age of the animals and chronicity of metal exposure (Figure 1D–I). Another key observation is the different pattern of α‐syn immunolabeling seen in the frontoparietal cortices of vanadium‐exposed animals. These varied from eclipse to spherical aggregates of variable sizes that were frequently adjacent to cells (Figure 2 and Figure S4). In some cases, α‐syn aggregates were associated with NeuN‐negative cells or naked nuclei; in some cases, α‐syn aggregates were also associated with NeuN‐negative cells or naked nuclei in degenerating neurons with cytoplasmic loss (Figure S4J). In some neurons, α‐syn immunoreactivity was apparent throughout the cell body and in the processes (arrow). In the frontoparietal cortices, we also observed abundant cytoplasmic and nuclear α‐syn inclusions. Double immunolabeling of α‐syn with glial cell marker (Iba1, GFAP) revealed functional gliosis as well as astrocytic and microglial activations at the sites of α‐syn immunoreactivity (Figures 3 and 4; Figure S4K,L). Quantitative analysis showed that microglial and astrocytic activation as well as α‐syn aggregation increase with increasing vanadium exposure in the frontoparietal cortices and dorsal hippocampal CA1 and CA3 regions (Figures 3 and 4).

### 3.5 Tau Immunolabeling and Cellular Localization

**FIGURE 5:**
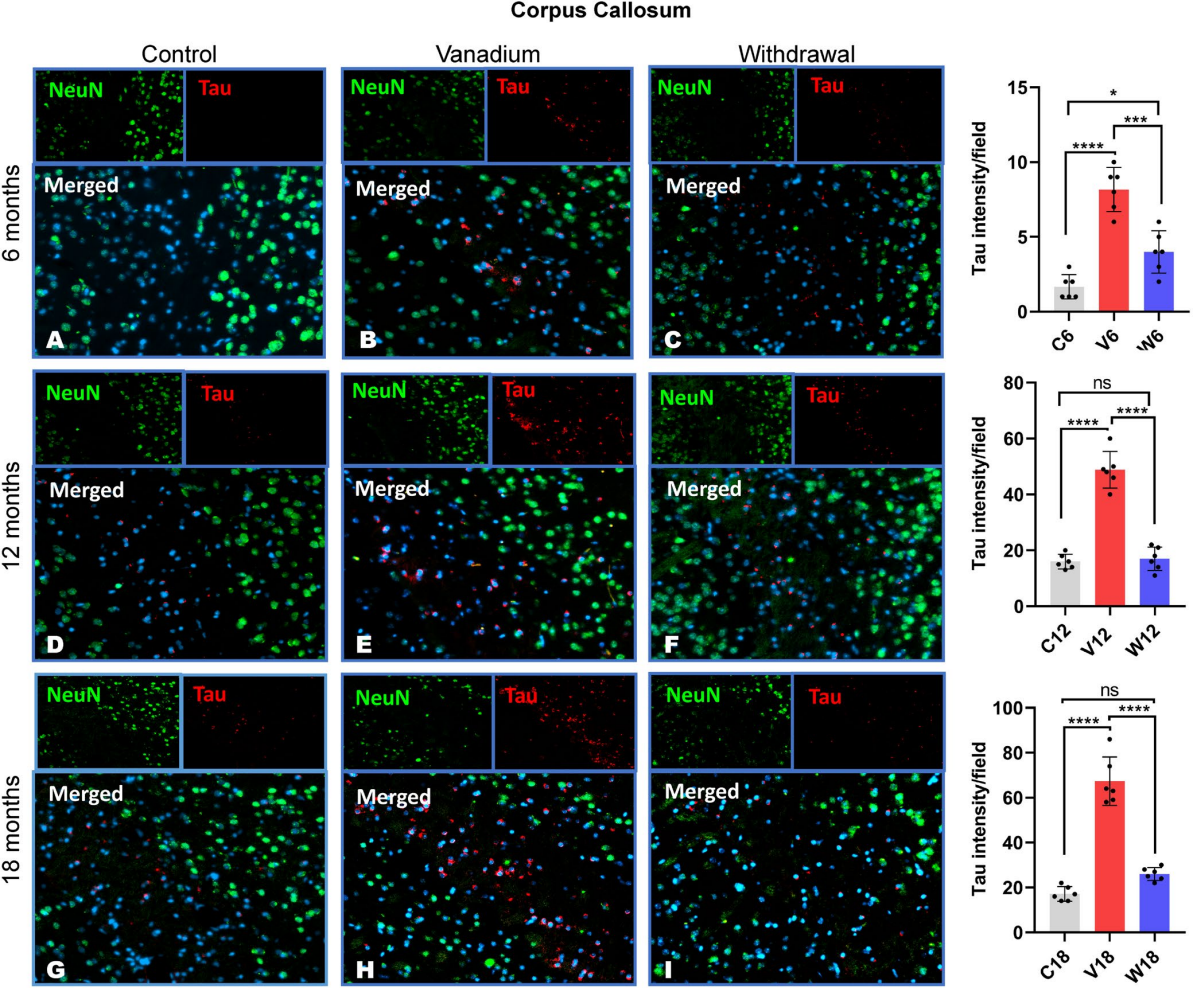
Tau phosphorylation and aggregation in the genu of corpus callosum of mice brains after 6 months (A–C), 12 months (D–F) and 18 months (G–I). Throughout the exposure periods, there were significant and progressive increases in the level of phosphorylated tau aggregates in the white tract of the corpus callosum in vanadium‐dosed groups (B, E, H) compared to age‐matched controls (A, D, G). However, a reduction in Tau immunoreactivity was observed in the withdrawal brains (C, F, I) compared to the vanadium‐treated brains. [*n* = 8 mice/group; intensity analysis = 6 mice; Scale bar: 50 μm; *p* < 0.05; Normality test (Shapiro–Wilk) = Corpus callosum (6 months: *p* value 0.8757, 12 months: *p* value 0.6270, 18 months: *p* value 0.0468) (*p < 0.05; ***p < 0.01; ****p < 0.0001)].

**FIGURE 6:**
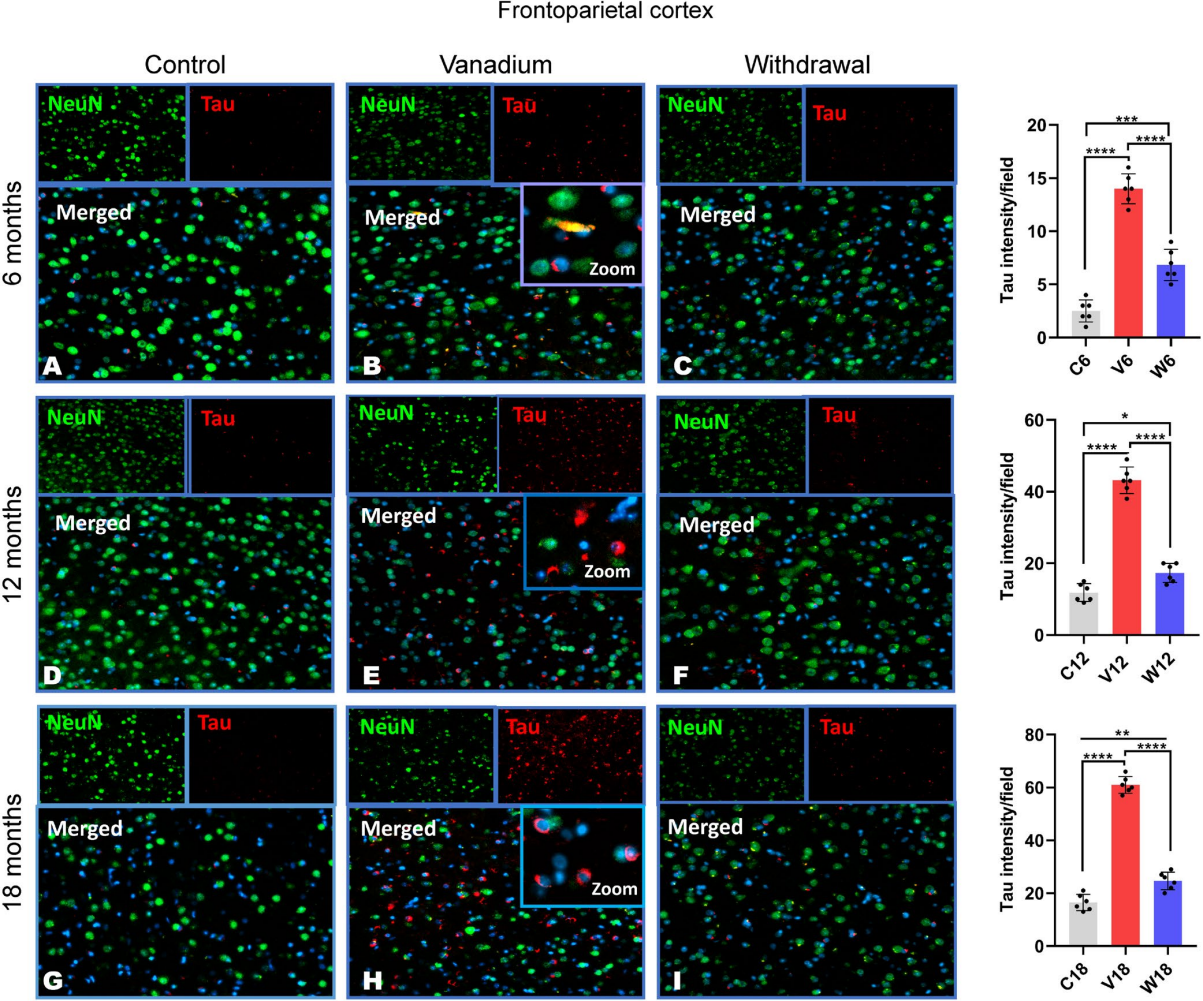
Tau phosphorylation and aggregation in the fronto‐parietal cortices (FPC) in mice brains after 6 months (A–C), 12 months (D–F) and 18 months (G–I). Throughout the exposure periods, there was a significant and progressive increase in the level of phosphorylated tau aggregates in the cortex of vanadium‐dosed groups (B, E, H) compared to age‐matched controls (A, D, G). However, a reduction in Tau immunoreactivity observed in the withdrawal brains (C, F, I) compared to the vanadium‐treated brains. [(*n* = 8 mice/group; intensity analysis = 6 mice; Scale bar: 50 μm; *p* < 0.05). Inset: Higher magnification; Normality test (Shapiro–Wilk) = FPC (6 months: *p* value 0.8043, 12 months: *p* value 0.8181, 18 months: *p* value 0.5703) (*p < 0.05; **p < 0.01; ***p < 0.001; ****p < 0.0001)].

Tau hyperphosphorylation and aggregation were detected in all the regions of the corpus callosum and parietal cortices (Figures 5 and 6). No phosphorylated tau immune‐reactivity/aggregation was detected in the hippocampus. The pattern of tau labeling revealed little or no immunoreactivity in the control animals (Figures 5 and 6A,D,G) relative to the vanadium‐exposed animal with numerous nuclei and cytoplasmic labeled hyperphosphorylated tau (Figures 5 and 6B,E,H). However, in the withdrawal animals, at every period of exposure (6, 12, and 18 months), there is a significant reduction of tau immuno‐reactivities (Figures 5 and 6C,F,I) when compared with those of the vanadium‐treated animals. Densitometric analysis of the immunostaining showed tau accumulation in the vanadium‐exposed brains increasing with the age of the animals and chronicity (dosage) of metal exposure (Figures S5 and S6). Co‐localization of tau immunolabeling with NeuN + DAPI in the parietal cortices showed tau accumulation in neuronal nuclei (Figure 6H see inset) but very few within the cytoplasm of the neurons, while in the cortices of old age (12–18 months) control animals, there was a relative lack of intracellular or nuclear tau immunoreactivity but occasionally detected labeled cells (Figure 6D,G). In addition, the severity of tau aggregation highly correlated with pronounced cell loss, observed in the cortices of treated animals. Quantitative analysis of the cell counts from the cortices of vanadium‐treated brains revealed decreased NeuN positive neurons in comparison with the age‐matched controls (Figure S6A: *p* < 0.05); this trend also increases with the severity of tau aggregation and the chronicity (dose) of vanadium exposure both in the corpus callosum and cortices (Figures S5 and S6). Also, in the parietal cortices of vanadium‐exposed animals are numerous intensively stained extracellular tau aggregates (Figure 6E, see inset). On the other hand, within the white matter of the corpus callosum of vanadium‐exposed animals were large numbers of intensely labeled nuclear tau aggregates (Figure 5E,H).

### 3.6 Aβ Immunolabeling and Cellular Localization

**FIGURE 7:**
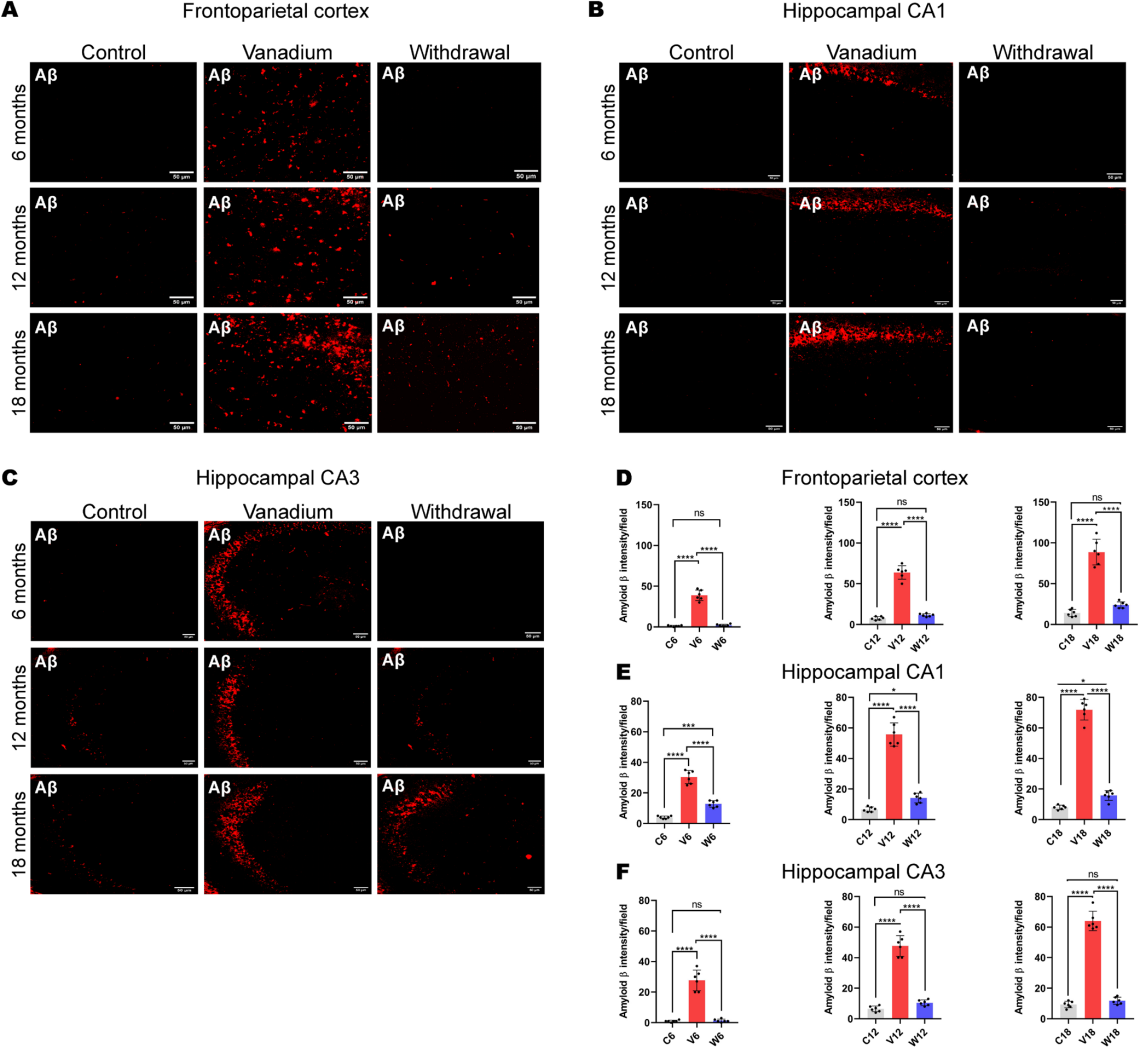
Immuno‐fluorescent staining showing amyloid beta (Aβ) aggregation/deposition in mice brains after 6–18 months of chronic vanadium treatment. Aβ accumulation was detected in the frontoparietal cortices, FPC (A), hippocampal CA1 region (B) and hippocampal CA3 region (C). At each period of exposure (6, 12, and 18 months), there was a significant increase in the level of Aβ deposition in the vanadium dosed group relative to control. However, a significant reduction was observed in the withdrawal brains relative to the vanadium animals. Densitometric quantification also confirmed increasing Aβ accumulation with advancing age and chronicity of vanadium exposure in the cortex (D), hippocampal CA1 (E) and CA 3 (F). [*n* = 8 mice/group; intensity analysis = 6 mice; Scale bar: 50 μm; *p* < 0.05; Normality test (Shapiro–Wilk) = FPC (6 months: *p* value 0.0356, 12 months: *p* value 0.0092, 18 months: *p* value 0.2071), CA1 (6 months: *p* value 0.9740, 12 months: *p* value 0.7442, 18 months: *p* value 0.0611), CA3 (6 months: *p* value 0.0064, 12 months: *p* value 0.2327, 18 months: *p* value 0.0116) (*p < 0.05; ***p < 0.001; ****p < 0.0001)].

Our results showed diffused intracellular accumulation of Aβ fibrils in the pyramidal neurons of the frontoparietal cortices and dorsal hippocampal CA1 and CA3 regions (Figure 7A–C). In contrast, control animals exhibited little or no amyloid accumulation, while withdrawal animals across all groups showed a significant reduction in Aβ levels compared to the vanadium‐treated animals over the exposure periods (6, 12, 18 months). Quantitative analysis of the Aβ immunoreactivities in the treated animals revealed progressive accumulation in the brain over months of exposure (from 6 to 18 months) and age of the exposed animals (Figure 7D,E).

**FIGURE 8:**
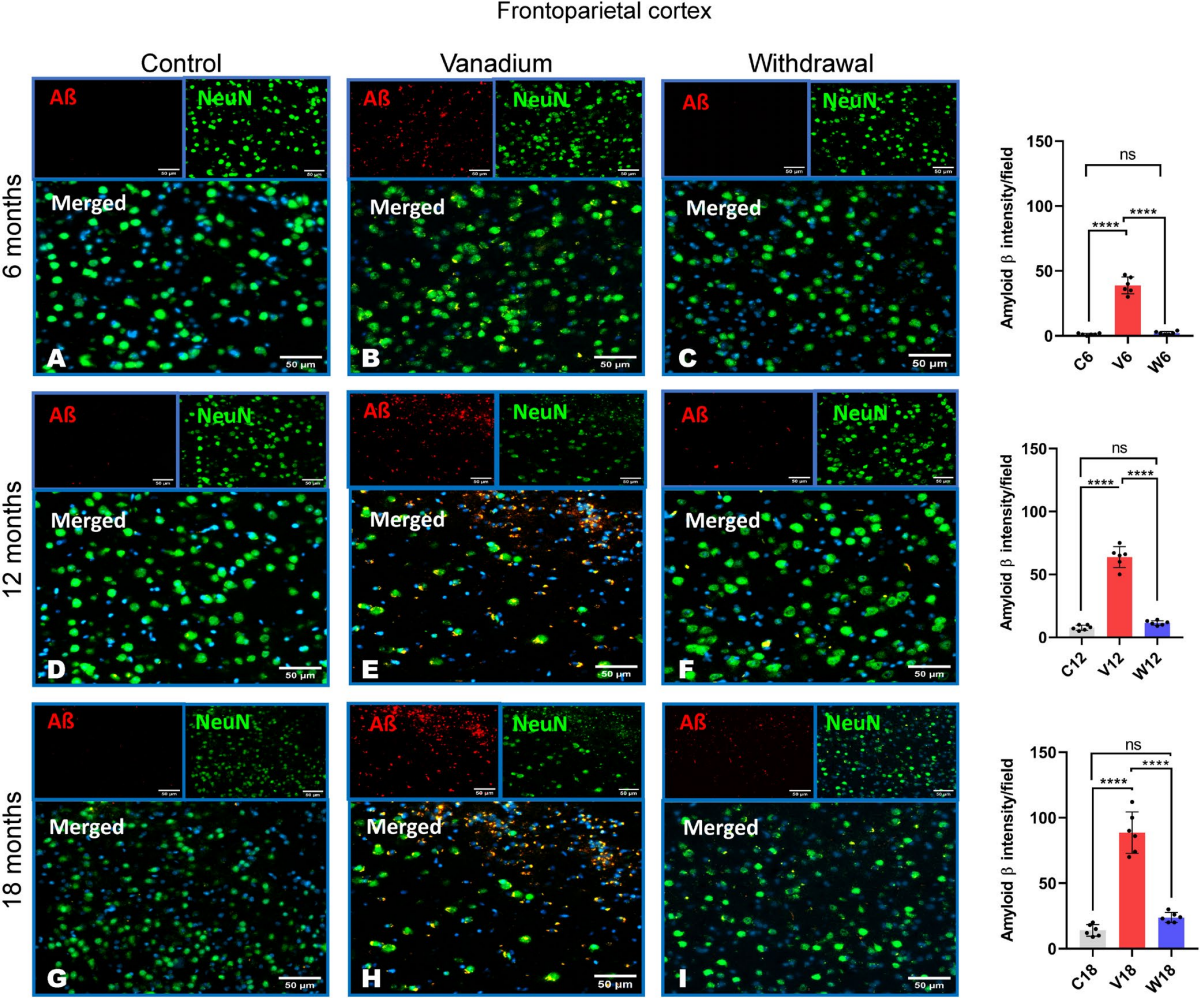
Amyloid beta (Aβ) and NeuN co‐labelling of the frontoparietal cortices after 6 months (A–C), 12 months (D–F) and 18 months (G–I) in mice brains. Controls (A, D, G) and withdrawal (C, F, I) groups displayed had very little to almost no Aβ deposits. Marked Aβ deposits were seen in vanadium exposed mice after 6–18 months of chronic vanadium exposure (B, E, H). Colocalization of Aβ immunolabeling with neuronal marker (NeuN) revealed diffuse Aβ deposits and pyramidal cell pathology including pyknosis, cytoplasmic loss and vacuolation. Quantitative analysis of intracellular Aβ aggregates intensities revealed significant levels of Aβ protein aggregation in vanadium exposed compared to control. Aβ aggregates intensities in withdrawal were similar to control group [*n* = 8 mice/group; intensity analysis = 6 mice; Scale bar, 50 μm; *p* < 0.05; Normality test (Shapiro–Wilk) = FPC (6 months: *p* value 0.0356, 12 months: *p* value 0.0092, 18 months: *p* value 0.2071) (****p < 0.0001)].

**FIGURE 9:**
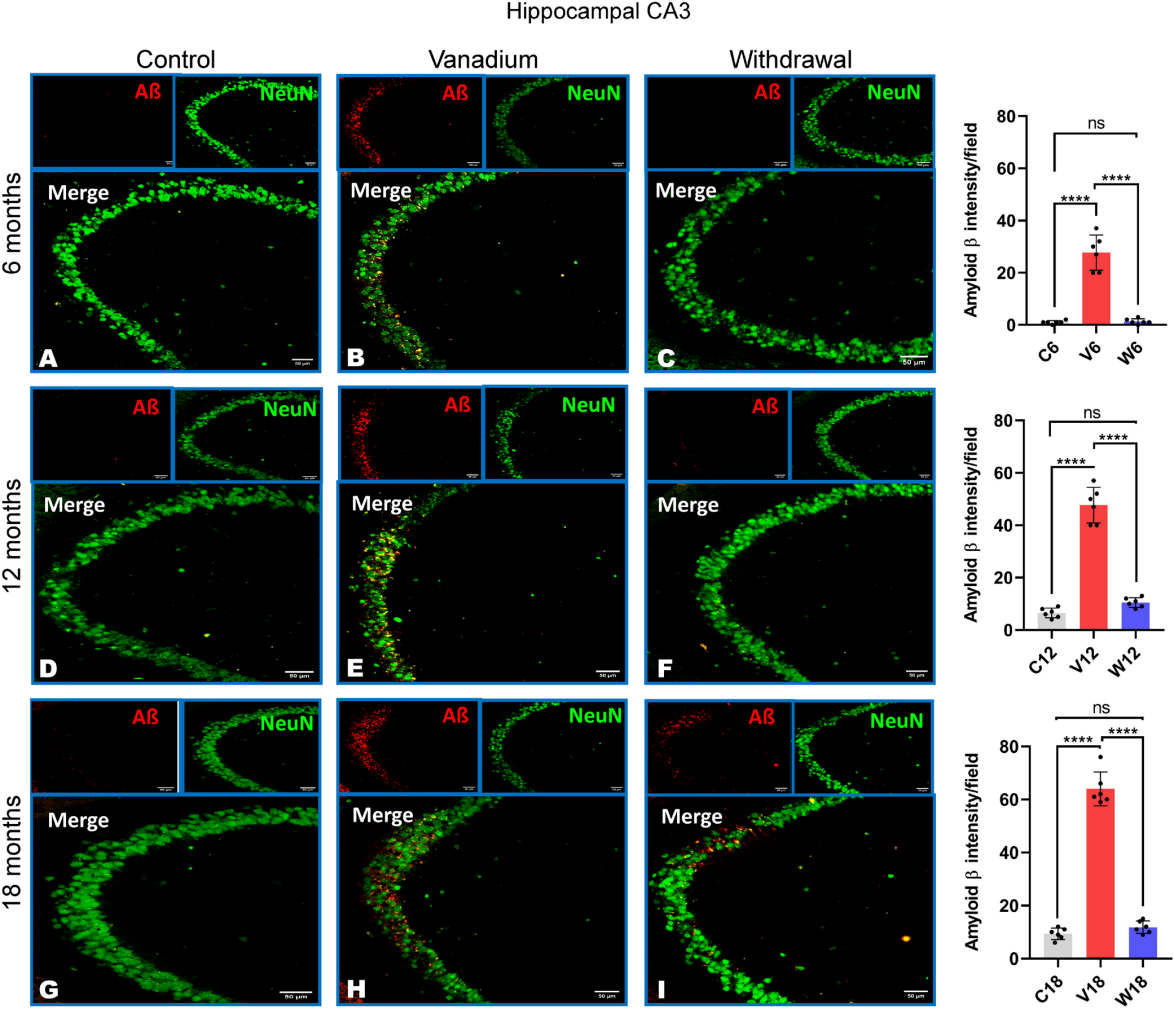
Amyloid beta (Aβ) and NeuN co‐labelling of the hippocampal CA3 after 6 months (AC), 12 months (D–F) and 18 months (G–I) in mice brains. Controls (A, D, G) and withdrawal (C, F, I) groups displayed very little to almost no Aβ deposits. Marked Aβ deposits were seen in vanadium‐exposed mice after 6–18 months of chronic vanadium exposure (B, E, H). Colocalization of Aβ immunolabeling with neuronal marker (NeuN) revealed diffuse Aβ deposits and pyramidal cell pathology including pyknosis, cytoplasmic loss, and vacuolation. Quantitative analysis of intracellular Aβ aggregate intensities revealed significant levels of Aβ protein aggregation in vanadium‐exposed compared to control. Aβ aggregate intensities in withdrawal were similar to the control group [*n* = 8 mice/group; intensity analysis = 6 mice; Scale bar, 50 μm; *p* < 0.05; Normality test (Shapiro–Wilk) = FPC (6 months: *p* value 0.0064, 12 months: *p* value 0.2327, 18 months: *p* value 0.0116) (****p < 0.0001)].

**FIGURE 10:**
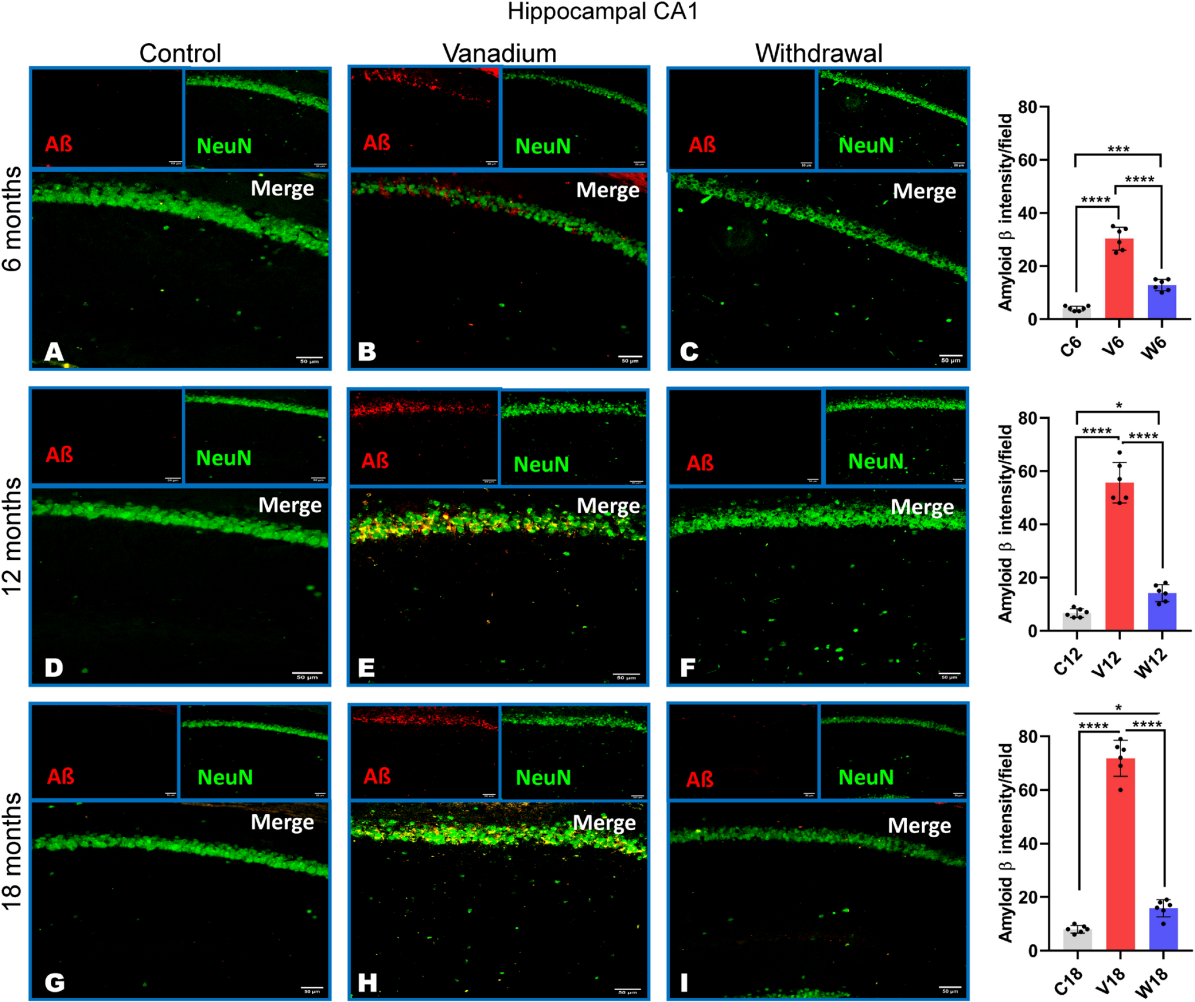
Intraneuronal amyloid beta (Aβ) and NeuN co‐labelling of the hippocampal CA1 after 6 months (A–C), 12 months (D–F) and 18 months (G–I) in mice brains. Controls (A, D, G) and withdrawal (C, F, I) groups displayed very little to almost no Aβ deposits. Diffused intraneuronal Aβ accumulation was seen in vanadium‐exposed mice after 6–18 months of chronic vanadium exposure (B, E, H). Colocalization of Aβ immunolabeling with the neuronal marker (NeuN) revealed pyramidal cell pathology including pyknosis and cytoplasmic loss at the anatomical site of Aβ deposition [(*n* = 8 mice/group; intensity analysis = 6 mice; Scale bar, 50 μm; *p* < 0.05) (*p < 0.05; ***p < 0.001; ****p < 0.0001)].

NeuN immunohistochemistry revealed neuronal pathology including hippocampal cell loss in both the parietal cortices (Figure 8) and dorsal hippocampal CA3 (Figure 9) and hippocampal CA1 regions (Figure 10) of vanadium‐exposed animals when compared to the controls. However, significant attenuation of cell loss was observed in the withdrawal animals at every period of vanadium exposure. Co‐localization of NeuN‐positive cells with Aβ immunoreactivities in the parietal cortices and dorsal hippocampus regions of vanadium‐treated animals revealed degenerating cells with Aβ deposits. Some cells in vanadium‐exposed brains exhibited extensive Aβ accumulation, while others showed little to none. Cells with intense Aβ immunoreactivities had disrupted cytoplasm and nuclei (Figure 8E,H). Neurons with low Aβ immunoreactivities maintained normal nuclear and cytoplasmic morphology (Figures 8, 9, 10). Quantitative analysis of cell counts indicated that cell loss and Aβ immunoreactivity increased with the duration of vanadium exposure (Figures 8, 9, 10 and Figure S7). Co‐localization of Aβ immunolabeling with NeuN‐positive cells revealed diffused intraneuronal Aβ accumulations and pyramidal cell pathology, including pyknosis, cytoplasmic loss, and vacuolation (Figure 8E,H).

## 4 Discussion

Vanadium exposure is associated with α‐syn aggregation in cellular elements of frontoparietal cortices and dorsal hippocampal CA1 and CA3 regions, which reveals the unique vulnerability of this brain region to vanadium‐induced synucleinopathy. This, to the best of our knowledge, is the first study to provide neuropathological evidence of α‐syn aggregation in the cortical and hippocampal regions after chronic vanadium neurotoxicity. A remarkable increase in the number of glial cells (activation) observed from the frontoparietal cortices and dorsal hippocampal CA1 and CA3 regions in this work highly correlates with the severity of α‐syn protein aggregation from these regions. This observation describes the role of glial activation and α‐syn aggregation in the pathogenicity of vanadium neurotoxicity (Wang et al. 2023). Although it is accepted that glial activation is triggered by neuronal damage caused by α‐syn aggregates, this study has shown that microglial activation may also exacerbate α‐syn pathology and the development of neurodegeneration. Under pathological conditions or aging, the capacity for α‐syn clearance by microglia declines owing to impairment of microglial phagocytic function, which further increases α‐syn accumulation in the brain (Pluvinage et al. 2019; Marschallinger et al. 2020). Apart from the impairment of protein degradation pathways due to aging (Choi et al. 2020), perpetual inflammation (Marschallinger et al. 2020; Scheiblich et al. 2021) from chronic assaults may also complement this impairment process and accelerate α‐syn oligomer accumulation. This observation might be accountable for the increasing α‐syn immunoreactivity/aggregation observed in the vanadium‐exposed animals with increasing age and chronicity of metal exposure. In age‐related disease like PD, microglia phagocytosis of α‐syn oligomers is significantly decreased in adult mice when compared with the young mice (Bliederhaeuser et al. 2016; Marschallinger et al. 2020). Also observed from the current work is the expression of α‐syn aggregates in the activated astrocytes, though at a level lower than that of the neurons, showing that astrocytes also play a crucial role in the α‐syn pathology in vanadium neurotoxicity. It has been reported that released α‐syn aggregates from neurons not only induce microglial activation but also activate astrocytes to exacerbate inflammation (Lee et al. 2010). This observation is supported by a previous study that showed that in postmortem PD brains, α‐syn aggregates are present not only in dopaminergic neurons but also in astrocytes (Wakabayashi et al. 2000; Braak et al. 2007).

Vanadium is a redox active metal that generates oxidative stress through Fenton‐type reaction. In this study, its interaction with α‐syn aggregation to generate oxidative stress is proposed as the mechanism that triggers neuronal toxicity and death. Although Afeseh Ngwa et al. (2009) reported that vanadium increased oxidative stress and activated the proapoptotic kinase PKCδ by caspase‐3‐dependent proteolysis in dopaminergic neurons, our lab has reported pathological evidence of vanadium oxidative damage in rodent brains (Folarin, Adaramoye, et al. 2017; Ladagu et al. 2023). In addition, PTEN‐induced kinase 1 (PINK−1) *Drosophila melanogaster* models of PD, exposed to chronic low doses of vanadium, exacerbated the existing motor deficits, reduced survival, and increased the production of reactive oxygen species (ROS) (Ohiomokhare et al. 2020). Although the precise mechanisms underlying these observations remain unclear and need to be further elucidated, oxidative stress and mitochondrial dysfunction likely played a role (Shvachiy et al. 2023).

Our results also provide evidence of tau hyper‐phosphorylation and aggregation in the cellular elements of the parietal cortices and white matter of the corpus callosum, suggesting chronic vanadium exposure as a potential risk for neurodegeneration. It should be, however, noted that the hippocampus did not show vulnerability as per tau expression. There are, however, accumulating evidences in literature showing vulnerability of these brain regions (Hippocampus, cerebral cortex and corpus callosum) to pathologic tau accumulation and neurodegeneration (O'callaghan et al. 2017; Detrez et al. 2019; Ferrer et al. 2020; Kubo et al. 2019; Fujimura and Usuki 2023). To the best of our knowledge, this is the first report of phosphorylated tau aggregation in the parietal cortices and corpus callosum following chronic vanadium neurotoxicity. However, we had reported high expression of tau in the brains of African Giant Rats caught in the wild in Nigeria, which incidentally had very high heavy metal contents (Olopade et al. 2023). We also observed that phosphorylated tau protein expression highly correlates with the degree of pyramidal cell loss in the parietal cortices after chronic vanadium neurotoxicity. Tau phosphorylation and aggregation are prerequisites for neurofibrillary tangles (NFTs) formation in tauopathies, including AD, and also constitute the molecular basis of cognitive impairment and neuronal loss associated with these diseases (John and Reddy 2021; Kim et al. 2024).

Immunoreactivity of extracellular tau aggregates observed in the cortices of vanadium‐exposed animals is suggestive of pathological spreading of tau aggregates in vanadium‐induced neurotoxicity. There is accumulating evidence showing that phosphorylated tau aggregates that leak from dying or degenerating neurons are subsequently released into the extracellular space via synaptic connections to form extracellular seed‐competent tau that further mediates inter‐neuronal propagation of tau to anatomically connected regions (Clavaguera et al. 2014; Miao et al. 2019; Ferrer et al. 2020).

Increased expression of nuclei labeled phosphorylated tau isomers (phosphorylated nuclear tau) observed both in the parietal cortices and corpus callosum white matter of the vanadium exposed animal is suggestive of the possible involvement of this nuclear tau isomer in the pathogenesis of vanadium induced neurotoxicity. Tau protein was originally localized in neuronal axons and isolated as a microtubule‐associated protein (Ait‐Bouziad et al. 2017; Antón‐Fernández et al. 2023); however, under pathological conditions, abnormal mislocation (TAU mis‐sorting) of tau may occur to other places, such as in the soma, the dendrites, and the nucleus of a cell (Li et al. 2018; Ittner and Ittner 2018; Antón‐Fernández et al. 2023) where they interfere with specific nuclear functions (Antón‐Fernández et al. 2023). This abnormal distribution of tau into sites where it is not normally localized has thus been suggested as a key step in the pathogenesis of tauopathies (Zempel and Mandelkow 2019; Zimmer‐Bensch and Zempel 2021).

In the current study, intraneuronal accumulation of Aβ deposits observed in the cortex and dorsal hippocampal regions suggests an early process preceding the extracellular accumulation of extracellular Aβ plaque in chronic vanadium neurotoxicity. This finding also proposes chronic vanadium exposure as a risk factor for AD. Dorado‐Martínez et al. (2024) reported neurofibrillary tangles, Aβ plaque accumulation in the vascular endothelium and pyramidal neurons, dendritic spine, and neuronal loss in the olfactory bulb and CA1 in mice after vanadium pentoxide inhalation. Studies have shown that in AD, Aβ starts accumulating inside neurons, causing neuronal damage before building up in the extracellular space (Moona et al. 2012; Okazawa 2021; Gallego Villarejo et al. 2022). The intracellular accumulation of Aβ observed in this study indicates the onset of Aβ pathology, which might accelerate disease progression and lead to extracellular Aβ plaque accumulation. Several in vivo and in vitro studies have proposed that intracellular Aβ aggregates can lead to the formation of extracellular fibrils (Takahashi et al. 2017).

This study also revealed a close correlation between intracellular Aβ accumulation and neuronal injury and loss in the parietal cortices and dorsal hippocampal regions. The precise causes of cell death in neurodegeneration are not fully understood; however, alterations in intracellular calcium (Ca^2+^) homeostasis have been implicated in neuronal dysfunction, excitotoxic cascades, and neuronal death (Harkany et al. 2000; Green et al. 2007; Ladagu et al. 2023). Recent research has implicated intracellular Aβ aggregates in disrupting axonal transport by attenuating mitogen‐activated protein kinase (MAPK) signaling pathways, which are necessary for neuronal synaptic transmission (Cruz et al. 2018).

The presence of a small number of aggregated tau and Aβ deposits in the aged control animals (12–18 months old) suggests age‐mediated neuronal vulnerability to oxidative stress and to amyloid‐protein aggregation. An age‐dependent accumulation of tau aggregation has been reported in a model (Aquino Nunez et al. 2022). Even without disease processes or oxidative damage, aging increases the brain's vulnerability to protein aggregation (Outeiro et al. 2024). Several studies have shown that many proteins become highly insoluble with age (Tanase et al. 2016; Groh et al. 2017; Lechler et al. 2017). However, it is still unclear how these insoluble misfolded protein aggregates contribute to disease processes.

The current study has demonstrated a relative reduction in vanadium‐induced neurotoxicity, as evidenced by improvements in neuronal pathology, neuro‐inflammatory injury, and various proteinopathies, including synucleinopathy, amyloid degeneration, and tau aggregation, following the cessation of vanadium exposure. This finding is consistent with our earlier studies, which reported significant elimination of vanadium from the brain, decreased oxidative damage, and reversal of memory loss after long‐term withdrawal from chronic vanadium treatment (Folarin et al. 2016; Folarin, Adaramoye, et al. 2017; Folarin, Snyder, et al. 2017). These effects may be attributed to reduced metal load and oxidative stress due to withdrawal of vanadium exposure, or repair processes compensating for early CNS damage (Woźniak et al. 2004; Stachowicz 2023). This may also imply that the brain exhibits a low potential for retaining absorbed vanadium after withdrawal (Folarin, Snyder, et al. 2017). Although transferrin is recognized as an active transporter of vanadium into the brain (Usende et al. 2016), the precise mechanism responsible for vanadium clearance from the brain needs to be further investigated. The slight increase in these proteinopathies with age after withdrawal group brains relative to control is still, however, worrisome as it indicates that some element of structural changes initiated by vanadium exposure in the developing brain still has long‐term consequences.

Several studies in the literature have reported the protective effects of synthetic antioxidants, plant natural flavonoids, and bio‐metal chelators against metal‐induced proteinopathy (Gugliandolo et al. 2019; Igado et al. 2020; Franklin et al. 2021; Fasae et al. 2021; Dash et al. 2021; Akash and Rehman 2021; Sienes Bailo et al. 2022; Jahić et al. 2020). Additionally, nearly complete withdrawal from toxic metal exposure over an extended period can alleviate metal‐induced neurodegeneration (Liu et al. 2012; Folarin, Snyder, et al. 2017). Therefore, it is crucial to raise awareness about the risks of hazardous metals, their safe use, and preventive measures during exposure to protect against their toxicity.

## Author Contributions

**O. R. Folarin:** methodology, investigation, data curation, writing – original draft, writing – review and editing, visualization, formal analysis. **F. E. Olopade:** conceptualization, writing – original draft, validation, visualization, writing – review and editing, supervision. **T. T. Gilbert:** writing – original draft, methodology, validation, visualization, writing – review and editing, software, data curation. **A. D. Ladagu:** methodology, visualization, writing – review and editing, validation, formal analysis, data curation. **P. I. Pires dos Santos:** methodology, validation, visualization, software, formal analysis, data curation. **O. A. Mustapha:** investigation, writing – original draft, methodology, validation, visualization, writing – review and editing, software, formal analysis, data curation. **L. Z. Kpasham:** writing – review and editing, validation, formal analysis, data curation, visualization. **J. O. Olopade:** conceptualization, investigation, funding acquisition, writing – original draft, methodology, validation, visualization, writing – review and editing, project administration, supervision, resources, formal analysis. **T. F. Outeiro:** conceptualization, investigation, funding acquisition, methodology, validation, visualization, writing – review and editing, formal analysis, project administration, supervision, resources.

## Conflicts of Interest

The authors declare no conflicts of interest.

### Peer Review

The peer review history for this article is available at https://www.webofscience.com/api/gateway/wos/peer‐review/10.1111/jnc.70082.

## Supporting information


Data S1.

## Data Availability

The data that support the findings of this study are available from the corresponding author upon reasonable request.
