# Supplementary material for: Chronic Vanadium Exposure Promotes Aggregation of Alpha‐Synuclein, Tau and Amyloid Beta in Mouse Brain
Source: J Neurochem. 2025 May 16;169(5):e70082. doi: 10.1111/jnc.70082 (PMC12082766; doi:10.1111/jnc.70082)
Supplement: Supplementary file 1 — Data S1. [file JNC-169-0-s001.pdf]

**Chronic Vanadium Exposure Promotes Aggregation of Alpha-Synuclein, Tau and Amyloid beta in Mouse Brain**

Folarin O. R[1], Olopade F. E.[2], Gilbert T. T.[3], Ladagu A. D.[3], Pires dos Santos P. I. [4], Mustapha, O.A[5]., Kpasham L. Z.[3], Olopade J. O.[3], Outeiro, T.F4,[6]

*[1] Department of Biomedical Laboratory Science, College of Medicine, University of Ibadan, Nigeria, [2] Department of Anatomy, College of Medicine, University of Ibadan, Ibadan, Nigeria, [3] Neuroscience Unit, Department of Veterinary Anatomy, Faculty of Veterinary Medicine, University of Ibadan, Ibadan, Nigeria, [4] University Medical Center Göttingen, Department of Experimental Neurodegeneration, Göttingen, Germany, [5]Department of Veterinary Anatomy, College of Veterinary Medicine, Federal University of Abeokuta, Abeokuta, Nigeria, [6]Translational and Clinical Research Institute, Faculty of Medical Sciences, Newcastle University, Framlington Place, Newcastle Upon Tyne, NE2 4HH, UK.*

**Correspondence:**

**Tiago F. Outeiro**

**Email:** touteir@gwdg.de

**Running Title: Vanadium Exposure Promotes Brain Proteinopathies**

## SUPPLEMENTARY FIGURES

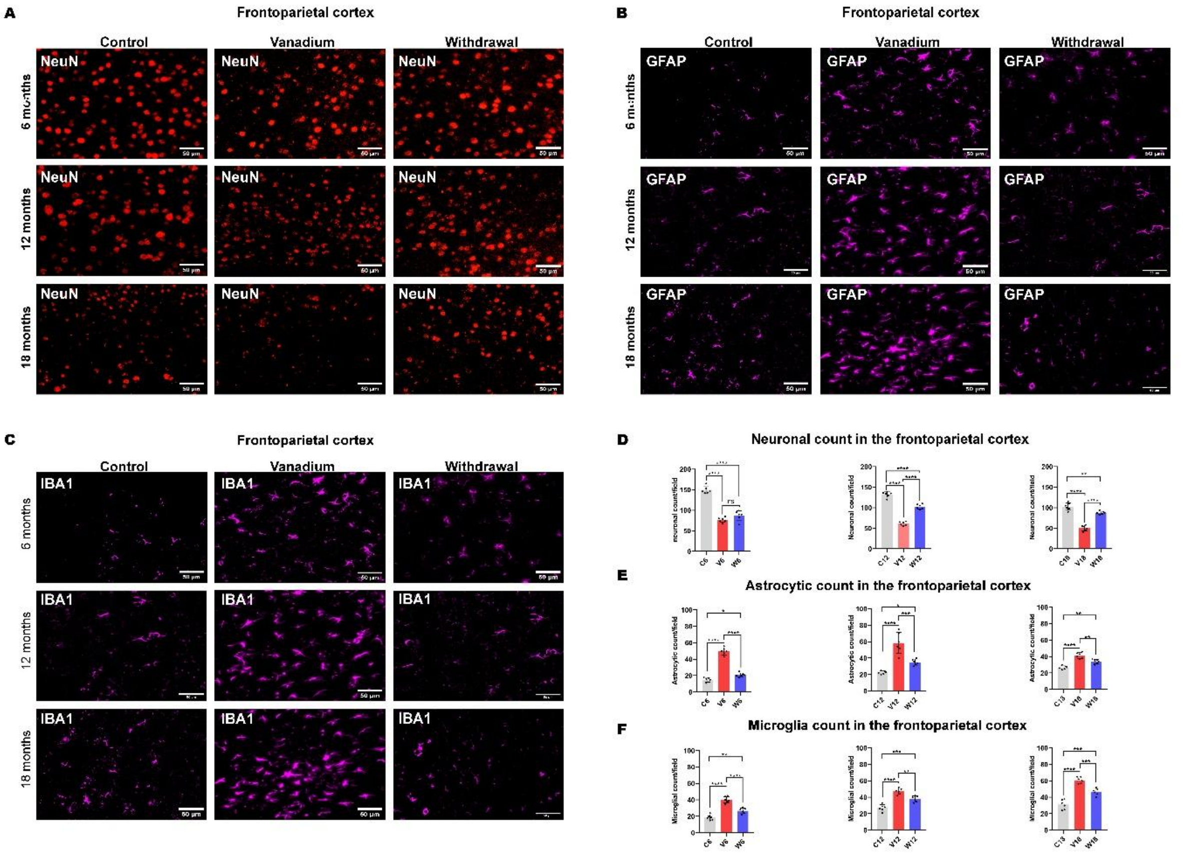


**Supplementary 1.** NeuN (A), GFAP (B) and IBA1(C) - Immunostained frontoparietal cortices (PFC) of control, vanadium-exposed, and withdrawal mice groups for 6 months, 12 months, and 18 months. (A) NeuN immunostaining showed evidence of neurodegeneration and pyramidal cell loss in the vanadium exposed brain relative to the control, while the withdrawal brain revealed mitigating effect when compared with the treated group. (B) Vanadium treatments for 6, 12, and 18 months revealed marked astrogliosis, characterized by hypertrophied soma and cytoplasmic processes, compared to age-matched controls with resting morphology. The withdrawal groups showed significant attenuation in astrocytic response relative to the vanadium-exposed groups. (C) Chronic vanadium treatment for 6 to 18 months revealed cells with markedly large, short, thickened processes in the vanadium-exposed brains compared to control. The withdrawal mice brains displayed less microglial immunoreactivity compared to the vanadium-exposed brains. (D) Quantitative analysis of neuronal count in the frontoparietal cortices showed progressive reduction of neurons with increasing exposure to vanadium into advanced age. (E) Quantitative analysis of astrocytic counts in the frontoparietal cortices confirmed progressive astrogliosis in vanadium exposed brains from 6 to 12 months, with subsequent attenuation from 12 to 18 months. (F) Quantitative analysis of microglia counts in the frontoparietal cortices showed progressive microgliosis with increasing exposure to vanadium into advanced age. [n = 8 mice/group; intensity

analysis = 6 mice; Iba1; Scale bar, 50 μm; $p < 0.05$. NeuN; Scale bar, 50 μm; $p < 0.05$). GFAP; Scale bar, 50 μm; $p < 0.05$; Normality test (Shapiro-Wilk) = Cortical neuronal count (6 months: p value 0.5494, 12 months: p value 0.2831, 18 months: p value 0.9888). Cortical astrocytic count (6 months: p value 0.9956, 12 months: p value 0.1257, 18 months: p value 0.8190). Cortical microglial count (6 months: p value 0.6165, 12 months: p value 0.7317, 18 months: p value 0.4090)].

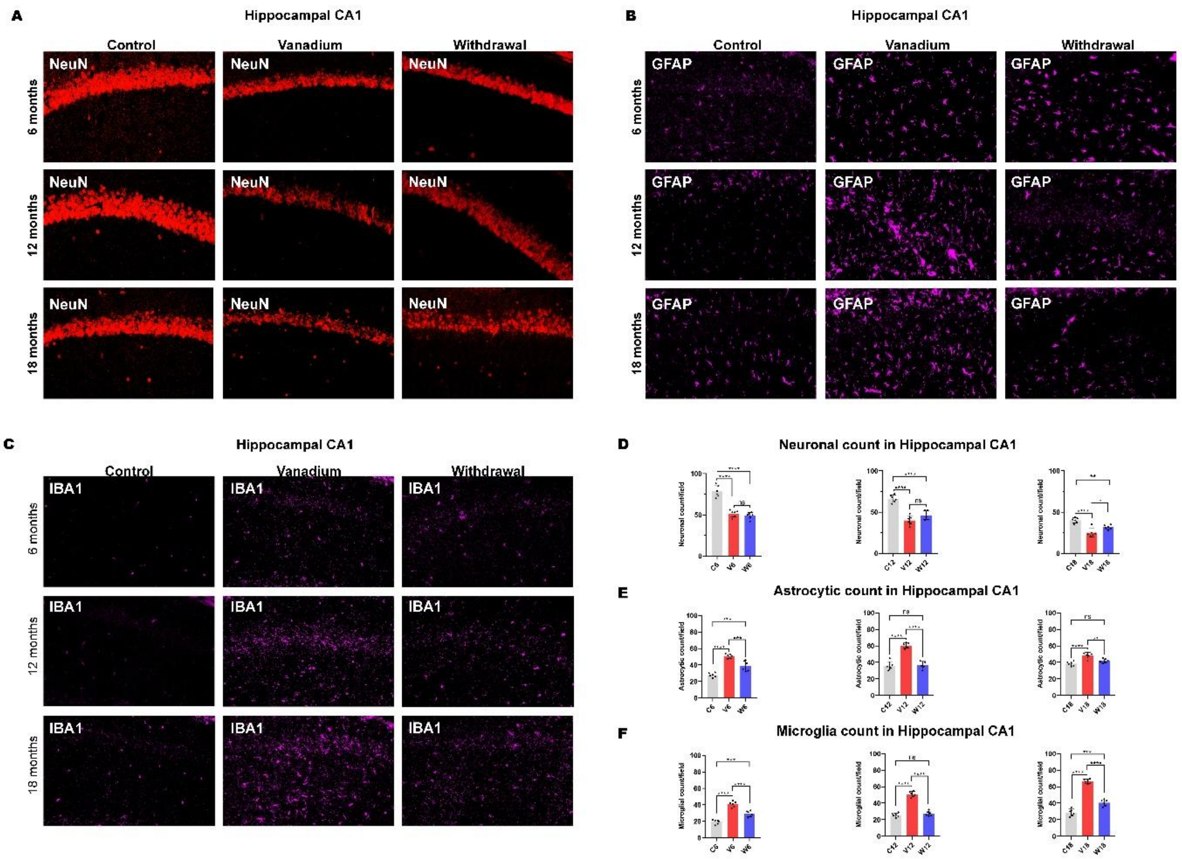


**Supplementary 2.** NeuN (A), GFAP (B) and IBA1 (C) - Immunostained Hippocampal CA1 pyramidal cells of control, vanadium-exposed, and withdrawal mice groups for 6months, 12months, and 18months. (A) NeuN immunostaining showed evidence of neurodegeneration and pyramidal cell loss in the vanadium exposed brain relative to the control, while the withdrawal brain revealed mitigating effect when compared with the treated group. (B) Vanadium treatments for 6, 12, and 18 months revealed marked astrogliosis, characterized by hypertrophied soma and cytoplasmic processes, compared to age-matched controls with resting morphology. The withdrawal groups showed significant attenuation in astrocytic response relative to the vanadium exposed groups. (C) Chronic vanadium treatment for 6 to 18 months revealed cells with markedly large, short, thickened processes in the vanadium-exposed brains compared to control. The withdrawal mice brains displayed less microglial immunoreactivity compared to the vanadium exposed brains. (D) Quantitative analysis of neuronal count in hippocampal CA1 showed progressive reduction of neurons with increasing exposure to vanadium into advanced age. (E) Quantitative analysis of astrocytes in hippocampal CA1confirmed progressive astrogliosis in vanadium-exposed brains from 6 to 12 months, with subsequent attenuation from 12 to 18 months. (F) Quantitative analysis of microglia counts in hippocampal CA1showed progressive microgliosis with increasing exposure to vanadium into advanced age. [n = 8 mice/group; intensity analysis = 6 mice; Iba1; Scale bar, 50 µm; $p < 0.05$). (NeuN; Scale bar, 50 µm; $p < 0.05$). (GFAP; Scale bar,

50 µm; $p < 0.05$). Normality test (Shapiro-Wilk) = CA1 neuronal count (6 months: p value 0.7294, 12 months: p value 0.4693, 18 months: p value 0.1365). CA1 astrocytic count (6 months: p value 0.8379, 12 months: p value 0.9560, 18 months: p value 0.4712). CA1 microglial count (6 months: p value 0.1327, 12 months: p value 0.8055, 18 months: p value 0.5340)].

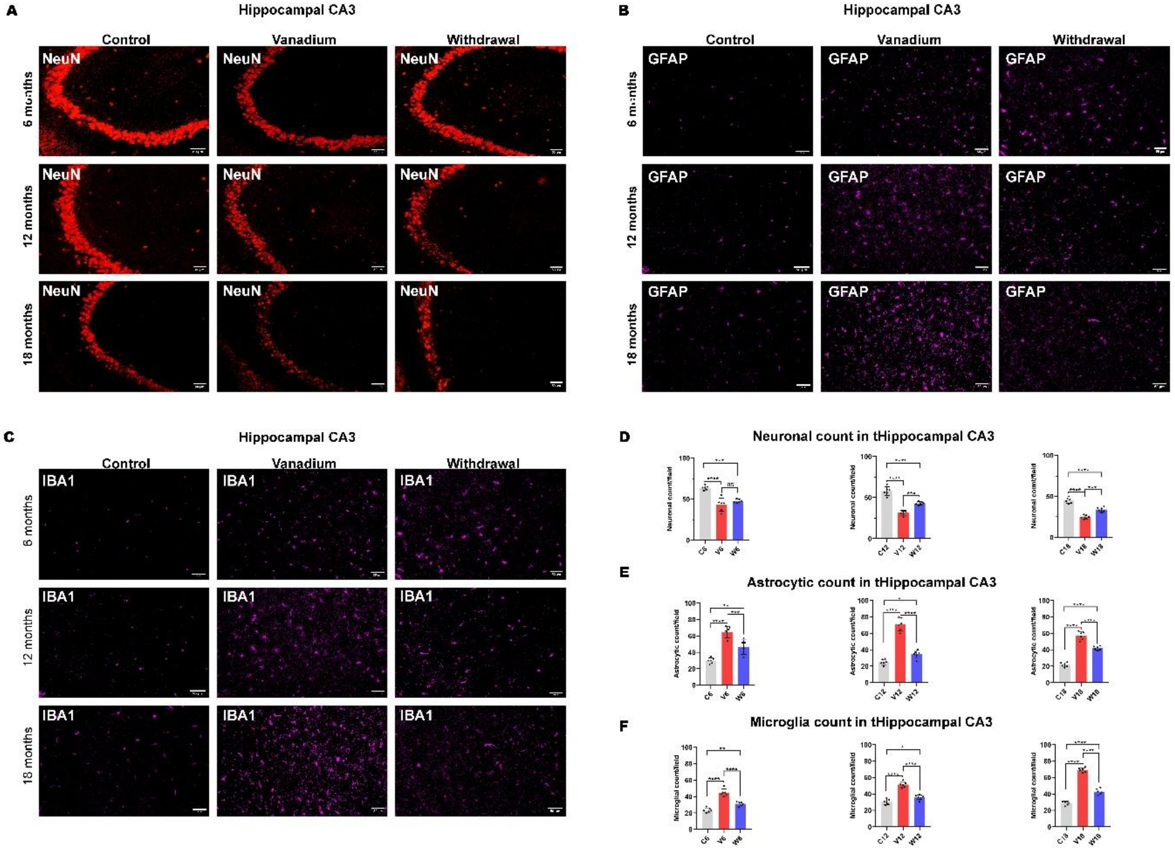


**Supplementary 3.** NeuN (A), GFAP (B) and IBA1 (C) - Immunostained Hippocampal CA3 pyramidal cells of control, vanadium-exposed, and withdrawal mice groups for 6months, 12months, and 18months. (A) NeuN immunostaining showed evidence of neurodegeneration and pyramidal cell loss in the vanadium exposed brain relative to the control, while the withdrawal brain revealed mitigating effect when compared with the treated group. (B) Vanadium treatments for 6, 12, and 18 months revealed marked astrogliosis, characterized by hypertrophied soma and cytoplasmic processes, compared to age-matched controls with resting morphology. The withdrawal groups showed significant attenuation in astrocytic response relative to the vanadium exposed groups. (C) Chronic vanadium treatment for 6 to 18 months revealed cells with markedly large, short, thickened processes in the vanadium-exposed brains compared to control. The withdrawal mice brains displayed less microglial immunoreactivity compared to the vanadium exposed brains. (D) Quantitative neuronal count in hippocampal CA3 showed progressive reduction of neurons with increasing exposure to vanadium with advancing age. (E) Quantitative astrocytic count in hippocampal CA3 revealed progressive astrogliosis in vanadium-exposed brains from 6 to 12 months, with subsequent attenuation from 12 to 18 months. (F) Quantitative microglial count in hippocampal CA3 showed progressive microgliosis with chronic exposure to vanadium. [n = 8 mice/group; intensity analysis = 6 mice; IBA1; Scale bar, 50 µm; $p < 0.05$). (NeuN; Scale bar, 50 µm; $p < 0.05$). (GFAP; Scale bar, 50 µm; $p < 0.05$); Normality test (Shapiro Wilk) =

CA3 neuronal count (6 months: p value 0.2560, 12 months: p value 0.6091, 18 months: p value 0.6598). CA3 astrocytic count (6 months: p value 0.6034, 12 months: p value 0.8108, 18 months: p value 0.9550). CA3 microglial count (6 months: p value 0.4144, 12 months: p value 0.7959, 18 months: p value 0.9756)]

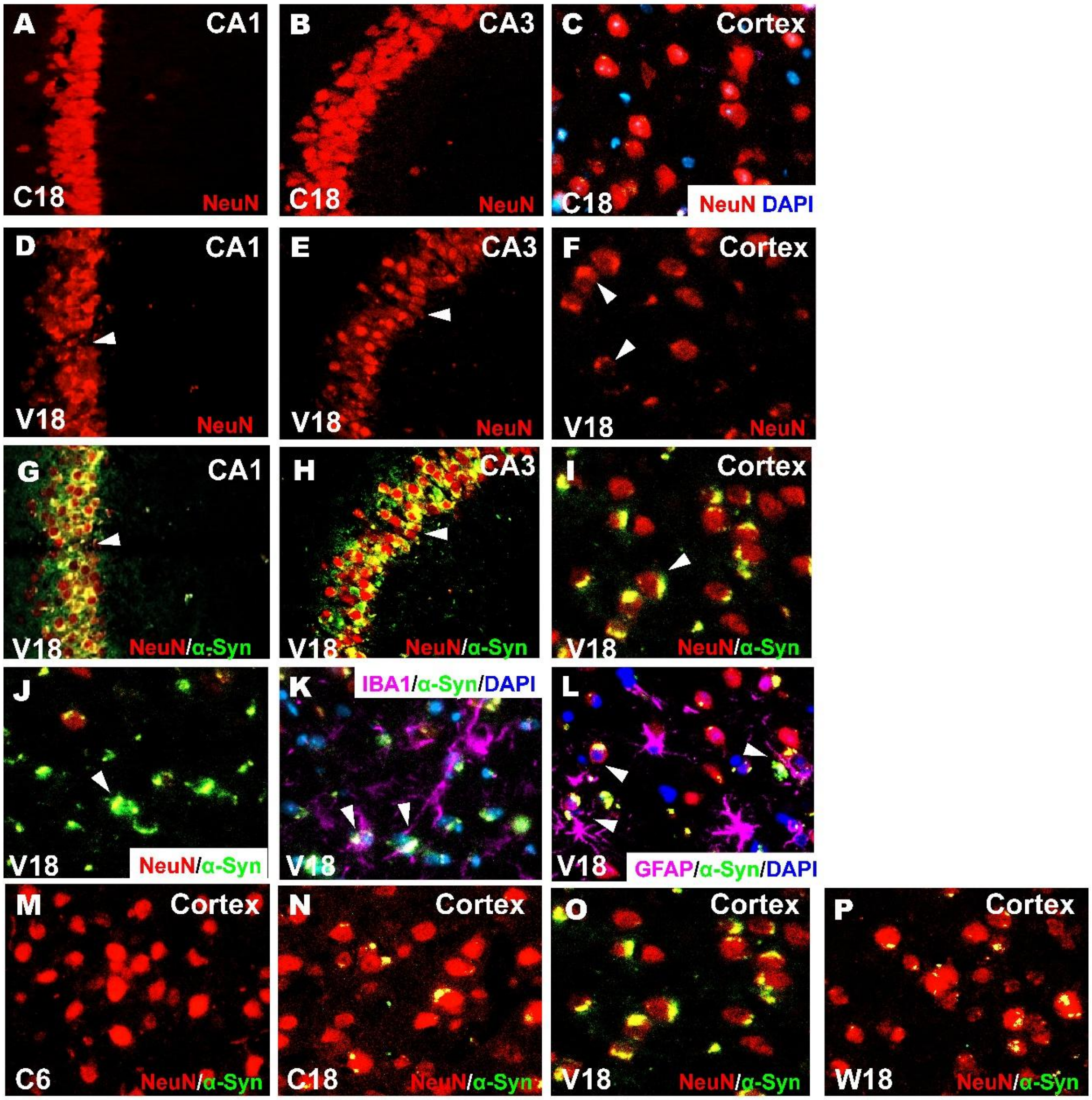


**Supplementary 4:** Immunofluorescence labeling of the hippocampal CA1, hippocampal CA3, and parietal cortical pyramidal cells. (A-C): NeuN immunostaining of neurons in control animals highlighting normal neuronal morphology. (D-F): Cellular cytotoxicity in the hippocampal and cortical pyramidal cells, characterized by cytoplasmic vacuolation and decreased in cellular density. (G-I): intraneuronal alpha-synuclein (α-syn) accumulation and aggregation in pyramidal neurons (arrows) of the hippocampal CA1 region, hippocampal CA3 region, and parietal cortices. (J): α-syn positive aggregates in the cortex in the absence of surrounding neuronal morphology, indicating neuronal loss (white arrows). (K, L): α-syn expressions in cortical microglia and astrocytes respectively. (M): immunolabeling of frontoparietal cortex in 6-month control showing

no cellular accumulation of α-syn, whereas (N) represents immunolabeling in an 18-month control animal with few α-syn expressions suggestive of α-syn with ageing. (O): intense α-syn accumulation and expressions in 18-month vanadium-exposed group, indicating that vanadium exacerbates α-syn aggregation with ageing. (P): immunolabeling in 18-month withdrawal animal, showing occasional α-syn labeling and attenuation of α-syn aggregation in the brain after withdrawal from vanadium treatment. (n = 8 mice/group; intensity analysis = 6 mice; Scale bar: 50 μm, $p > 0.05$).

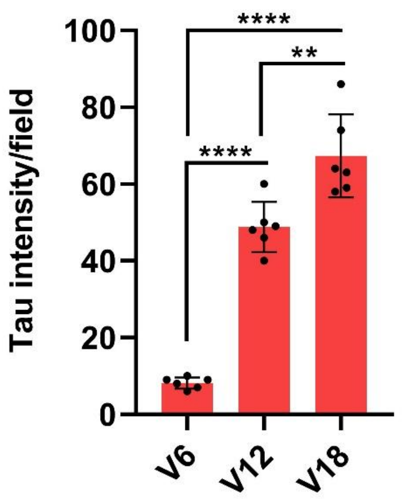


**Supplementary 5:** Densitometric quantification of Tau immunosignalling in the corpus callosum of vanadium dosed group from 6 to 18 months. A significant increasing trend of tau immunoreactivity with chronicity of vanadium exposure. [n = 8 mice/group; intensity analysis = 6 mice; $p<0.05$; Normality test (Shapiro-Wilk) = Corpus callosum: p value 0.0398].

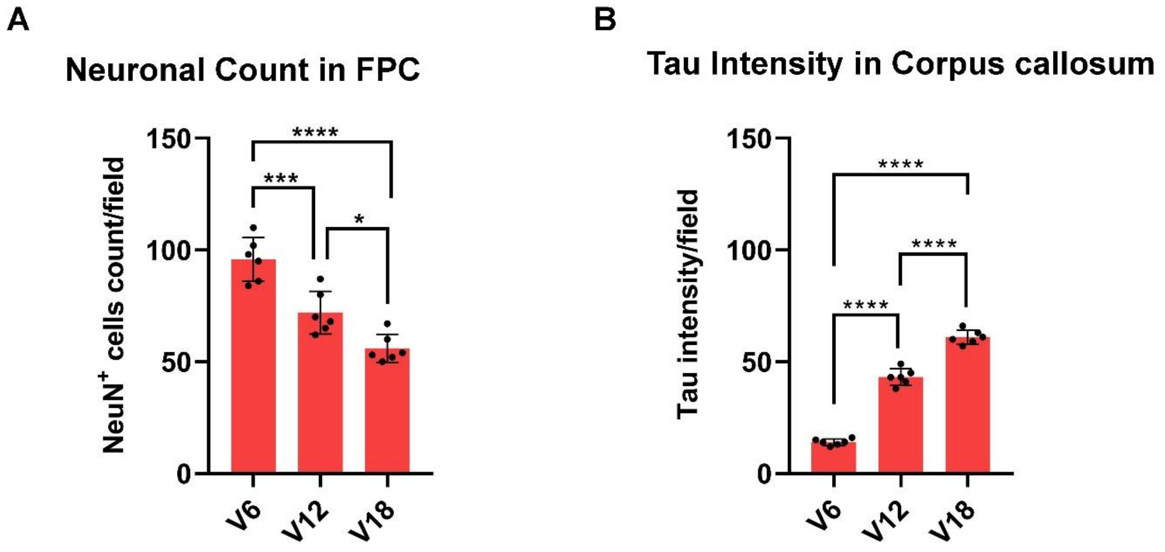


**Supplementary 6**. Densitometric quantification of (A) NeuN positive cells in the frontoparietal cortex, FPC and (B) Tau positive cells in the corpus callosum of vanadium dosed group from 6 to 18 months. A significant and progressive decrease in neuronal cell counts (A) with a progressive increase in tau immunoreactivity (B) were noted following chronic vanadium exposure. [n = 8 mice/group; intensity analysis = 6 mice; p<0.05; Normality test (Shapiro-Wilk) = Corpus callosum (NeuN: p value 0.3565, Tau: p value 0.5998)].

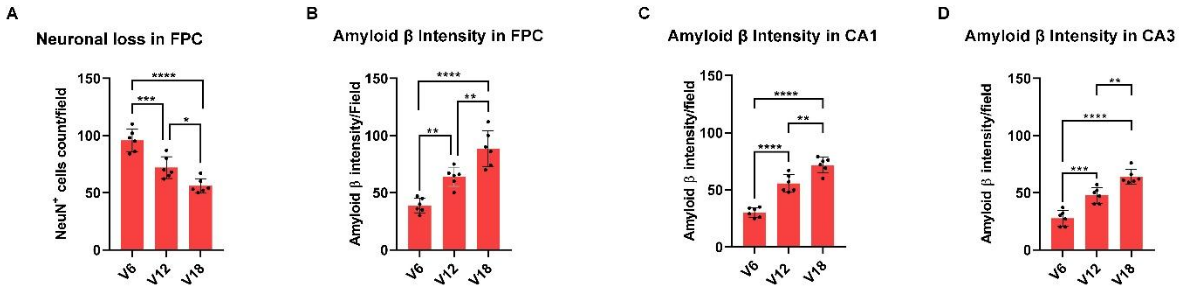


**Supplementary 7**: Densitometric quantification of neuronal and amyloid beta (Aβ) immunosignalling in vanadium dosed groups from 6 to 18 months. There was a significant and progressive decrease in neuronal cell counts (A), with a progressive increase in tau immunoreactivity both in the fronto-parietal cortices, FPC (B), hippocampal CA1 (C) and CA3 (D). [n = 8 mice/group; intensity analysis = 6 mice; $p<0.05$. Normality test (Shapiro-Wilk) = Neuronal lost: p value 0.3565; Aβ (FPC: p value 0.1967, CA1: p value 0.9556, CA3: p value 0.5998)].
